# An Open-Source 3D-Printable Platform for Testing Head-Fixed Cognitive Flexibility in Rodents

**DOI:** 10.1523/ENEURO.0364-24.2024

**Published:** 2025-01-24

**Authors:** Mark H. Cristino, Alexander C. Mitchell, Maya Preibisz-Kamat, Peyton Shea Fletcher, Timothy J. Spellman

**Affiliations:** ^1^Department of Biomedical Engineering, University of Connecticut, Glenbrook, Storrs, Connecticut 06269; ^2^Department of Neuroscience, University of Connecticut School of Medicine, Farmington, Connecticut 06030

**Keywords:** behavioral platform, cognitive flexibility, multisensory integration, open-source

## Abstract

The study of the neural circuitry underlying complex mammalian decision-making, particularly cognitive flexibility, is critical for understanding psychiatric disorders. To test cognitive flexibility, as well as potentially other decision-making paradigms involving multimodal sensory perception, we developed FlexRig, an open-source, modular behavioral platform for use in head-fixed mice. FlexRig enables the administration of tasks relying upon olfactory, somatosensory, and/or auditory cues and employing left and right licking as a behavior readout and reward delivery mechanism. The platform includes hardware and software components that are customizable, scalable, and portable, supporting a variety of behavioral assays. Using FlexRig, we established a head-fixed task to model attentional set-shifting, offering a new tool for neuroscience research that enhances the capacity for investigation of cognitive processes and their neural substrates, with broad applications in translational neuroscience.

## Graphical abstract

**Figure:**
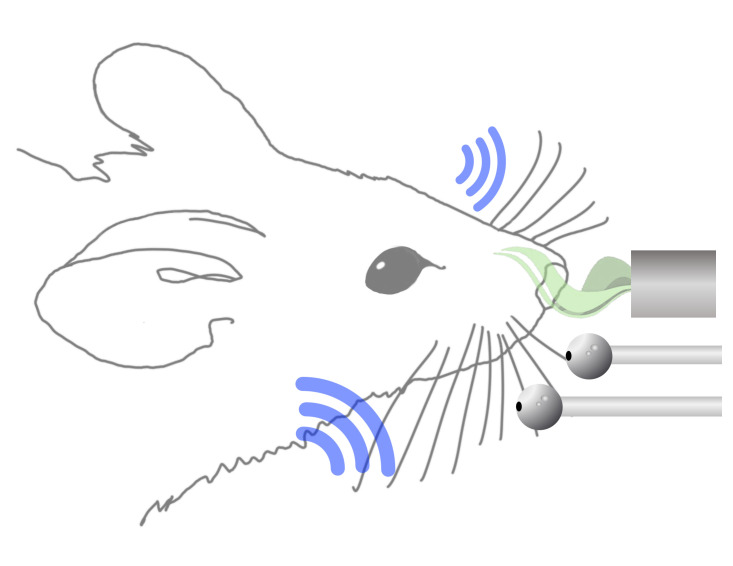
Visual Abstract

## Significance Statement

This manuscript details the development and operational guidance of FlexRig, a novel, open-source behavioral testing apparatus designed for administering tasks involving multiple sensory modalities, including attentional set-shifting tasks, in head-fixed mice. The platform's design emphasizes modularity, cost efficiency, and ease of replication, facilitating its adoption across various research laboratories. Although this paper does not present neural recordings, the FlexRig apparatus is optimized for integrating such technologies in future studies. This tool is expected to advance research into the neural bases of mammalian cognition by enabling controlled, reproducible behavioral experiments.

## Introduction

A core goal of systems neuroscience is to model the neural basis of behavior. Advances in experimental tool development over the past several years have enabled researchers to record and modulate the activity of large numbers of neurons in vivo during both innate and learned behaviors, with ever greater temporal, spatial, and cell-type specificity (Wiegert et al., 2023). To meet the challenge of performing rigorous and reproducible recording in head-fixed animals, we have developed and shared multiple open-source behavioral testing platforms in recent years. Some of these efforts have succeeded in demonstrating reproducibility in behavioral phenotyping across labs and even across international borders (Aguillon-Rodriguez et al., 2024). Through iteratively building upon these shared methods, the field of behavioral neuroscience has undergone both proliferation and convergence. Proliferation has come in the form of multiple specific behavioral platforms, tailored to the unique needs of each experiment, which often requires custom hardware configuration that limits the adoption of universal plug-and-play solutions. But behavioral methods have also seen convergence on certain core hardware and software solutions, which serve as infrastructure on which rodent behavioral testing platforms are built. From a hardware standpoint, these include the use of optical breadboards and mounting systems to position rig components, low-current solenoids and linear actuators to control water spouts, capacitive-touch sensors to detect licks, and air-supported trackballs to read out locomotion. From a software standpoint, this has included the use of Arduino and Raspberry Pi microprocessors and Python and MATLAB platforms to administer and log the tasks. The behavioral platform presented here builds upon this growing body of work, incorporating this hardware and software infrastructure into a novel design that offers both a specific solution to an experimental requirement (the need to administer complex multimodal sensory cues) while adopting a design that allows for use with numerous experimental paradigms going forward.

A set of behaviors that has garnered special focus, particularly in mammalian systems, are so-called flexible behaviors, also referred to as “behavioral” or “cognitive flexibility” (Hamilton and Brigman, 2015). In a cognitive flexibility task, a subject first learns to make a decision, typically motivated by an appetitive reward, such as food or water. At a time chosen either randomly or by the experimenter, the task contingency changes, so that the subject must change strategy in order to obtain the same reward. These tasks are of particular interest in translational neuroscience, because the ability to efficiently execute such a strategy change is associated with the healthy functioning of the prefrontal cortex (Milner, 1963; Friedman and Robbins, 2022), a uniquely mammalian brain region, and one phylogenetically closely linked with human evolution (Laubach et al., 2018). Deficits in cognitive flexibility are associated with damage to the prefrontal cortex, as well as a broad range of psychiatric conditions, including schizophrenia, attention deficit/hyperactivity disorder, anorexia nervosa, and major depression (Grant and Chamberlain, 2023). A classic test of cognitive flexibility is the Wisconsin Card-Sorting Test (Grant and Berg, 1948), in which a subject is given a deck of cards with objects on them that vary by number, color, and shape, and the subject is asked to sort the deck into piles. Each time the subject lays a card on a pile, they are told whether they have sorted the card correctly, but they are not told the sorting rule (which card feature is the relevant one). After successful learning of the relevant sorting rule, the rule is covertly changed, so that the previously relevant object feature becomes irrelevant and a different feature becomes newly relevant for sorting. The shifting of attention from one stimulus feature to another required to solve this task is called “attentional set-shifting.”

To elucidate the neural bases of cognitive flexibility, we set out to adapt an analogous task for use in mice. Traditionally, attentional set-shifting is assayed in rodents using an *ad libitum* moving paradigm, in which mice or rats dig for food rewards from bowls filled with digging media that vary by odor and texture (Birrell and Brown, 2000; Bissonette et al., 2008). This approach requires the animal to attend to either olfactory or somatosensory cues, two highly salient sensory modalities in rodents, to locate the reward. Because multiple methods in behavioral neuroscience require a head-fixed preparation, including certain in vivo imaging modalities, we sought a head-fixed behavioral paradigm.

While multiple research groups in recent years have developed and shared open-source behavioral testing platforms for use with head-fixed rodents and in vivo recordings, none satisfied the criteria needed to administer the attentional set-shifting paradigm used here. The proliferation of open-source hardware for use in behavioral neuroscience has yielded some highly useful contributions, such as systems for head fixation (Groblewski et al., 2020; Hughes et al., 2020; Weaver et al., 2023), and some have been designed for passive pavlovian conditioning paradigms (Hegedüs et al., 2021), while others have incorporated motor readouts for operant conditioning (Gordon-Fennell et al., 2023). Among platforms that incorporate variable stimulus presentation and motor responses, many of these systems leverage the visual system (Ozgur et al., 2023; Aguillon-Rodriguez et al., 2024), including virtual reality-based navigation systems (Lacefield et al., 2023; Cano-Ferrer et al., 2024). However, to ensure the closest possible analog to the *ad libitum* moving set-shifting paradigm on whose prior body of work we intended to build, we sought instead to leverage the whisker somatosensory and olfactory systems. Open-source platforms capable of administering whisker somatosensory cues (Guo et al., 2014) and olfactory cues (Han et al., 2018) have also been published, but these are single-modality systems, whereas the set-shifting task requires multisensory capability. For these reasons, and to facilitate replicability of findings and collaboration between labs, we developed an open-source behavior platform, which we call FlexRig.

The system we designed and built is modular, cost-effective compared with commercially available testing systems, relatively simple to assemble and use, scalable, and adaptable to a wide range of head-fixed cognitive and behavioral testing paradigms. The system is also compact and portable, in that its self-contained design makes for easy transport and reinstallation in new environments, such as an imaging table. The name refers to the suitability of the system for testing cognitive flexibility, and also to the fact that the system can be used to test a wide range of mouse behaviors involving whisker, odor, and/or auditory sensation and driven by motivation for liquid rewards.

## Materials and Methods

**Figure 1.**
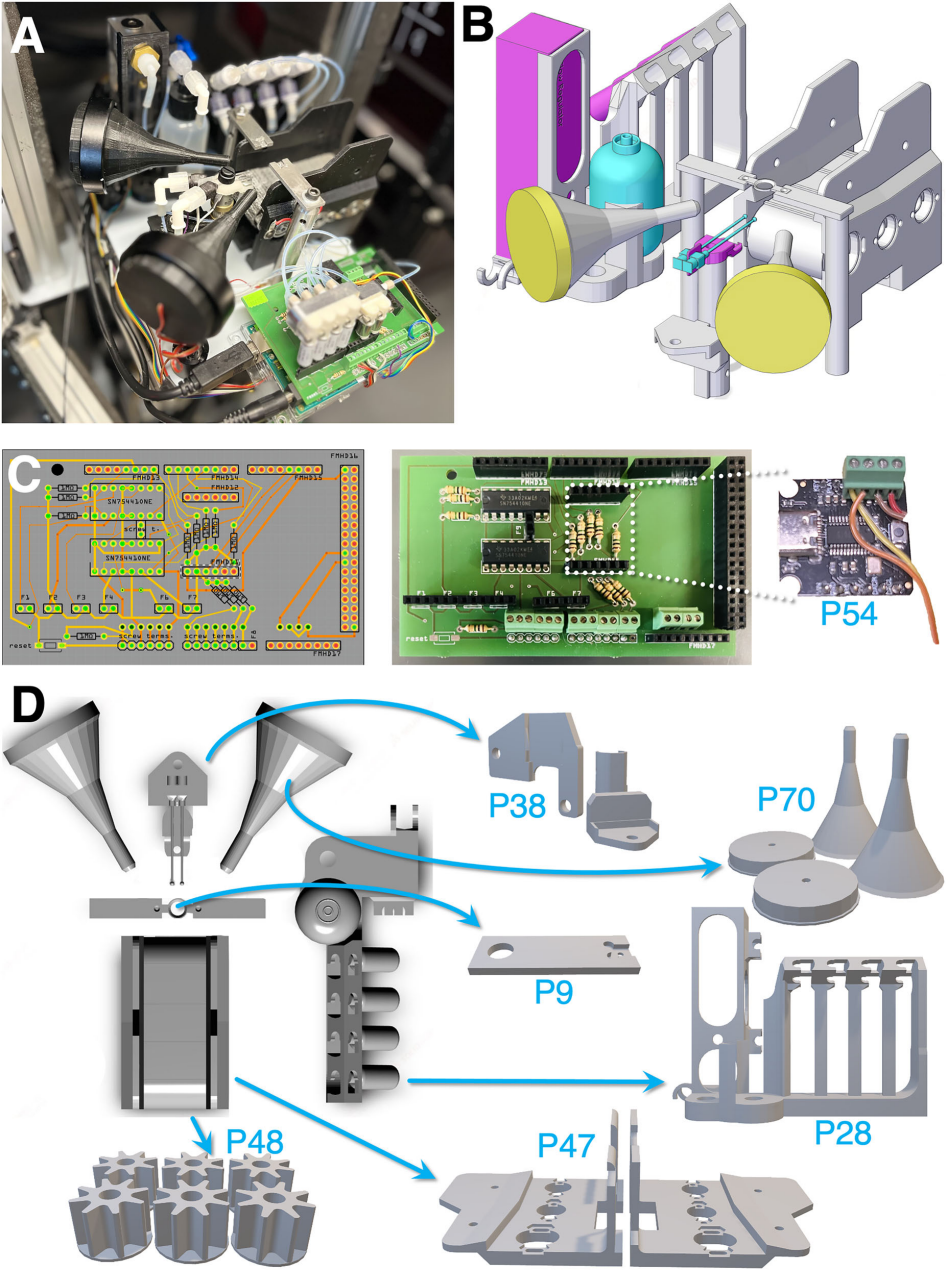
Rig design and custom part schematics. ***A***, Photograph of a completed rig. ***B***, Rig schematic. ***C***, Left, Arduino PCB shield wiring diagram. Right, Photograph of assembled PCB shield and sound card. ***D***, Schematic top view and breakout views of 3D-printed components. P38, A set of adapters for mounting left and right lickspouts to a vertical translating post and an XY translating stage (lickSpoutPostHolder.stl); P70, A set of funnels for directing a concentrated low-frequency sound wave toward the vibrissae (speakerFunnel.stl); P9, An adapter for mounting the animal's head plate to a 3″ vertical post (headbar.stl); P28, An adapter for mounting air and water routing components, including a water bottle, a set of four odorant bottles, and a set of two 3-way stopcocks (airWaterHolder.stl); P48, A set of aluminum treadmill sprockets for supporting and rotating a conductive treadmill (treadmillSprockets.stl); P47, A base for holding the treadmill (treadmillBase.stl).

**Figure 2.**
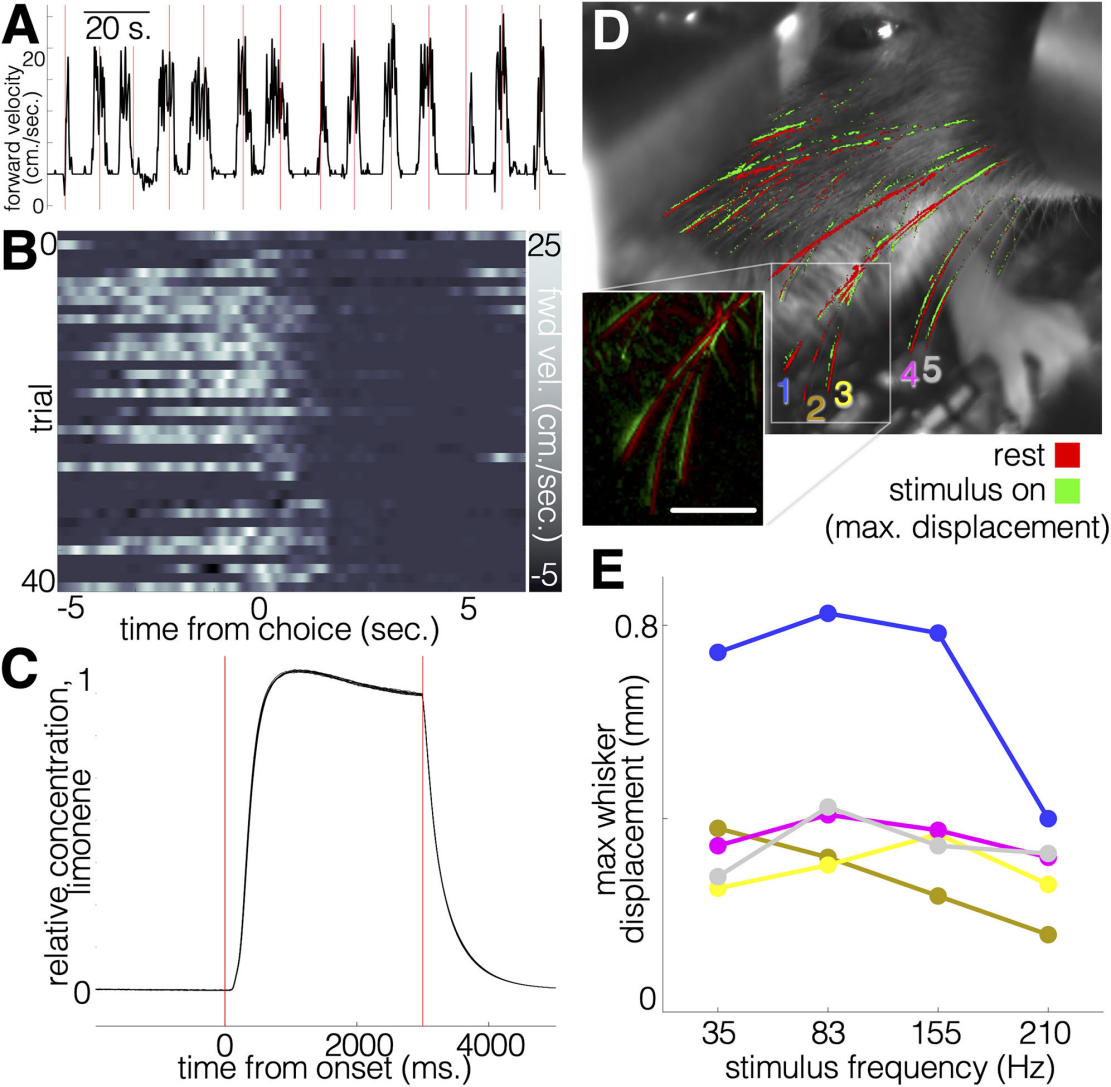
Interface between animal and hardware. ***A***, Example trace of spontaneous treadmill running from a single animal. ***B***, Treadmill running from the session shown in ***C***, arranged as per-trial heat plots. Animals generally begin running in anticipation of trial onsets and stop running during lick responding and water consumption. ***C***, Onset and offset of odorant concentration in the nose area, as measured with an MQ-3 sensor (10 sweeps). Latency between the odorant-on command signal and positive detection slope is 115–120 ms. ***D***, Soundwave-mediated whisker vibration. Superimposed mean video frames from a 10 s baseline (whiskers in red) and 2 s whisker vibration (whiskers in green) recording. Stimulus presented was 155 Hz, and camera framerate was 30fps. Stimulus intensity was ∼80 dB at the whiskers. ***E***, Maximum whisker displacement across a range of stimulus frequencies for the whiskers labeled in ***A***.

**Figure 3.**
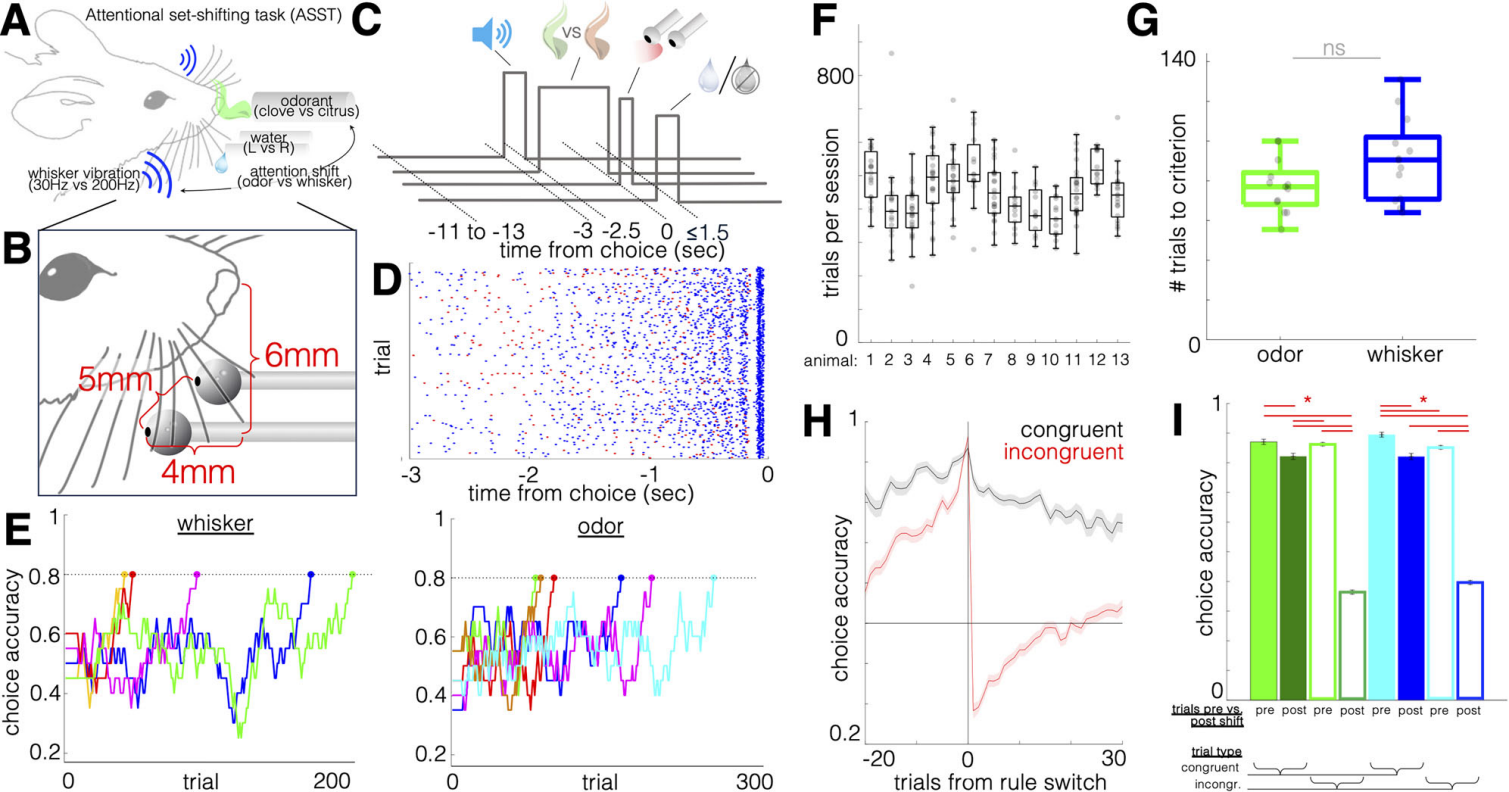
Training of a cross-modal attentional set-shifting task in mice. ***A***, Schematic of the head-fixed attentional set-shifting task. Mouse must periodically shift attention between compound whisker + odor stimuli to locate water. ***B***, Positioning of lickspouts relative to the mouse's nose. ***C***, Trial schematic. ***D***, Raster plot of licks toward (blue) and away from (red) the animal's final choice (time 0). Stimuli begin 2.5 s before the onset of the 1.5 s response window. Anticipatory licking accelerates through the stimulus presentation epoch and takes on an 8–10 Hz oscillatory pattern (bottom). ***E***, Response accuracy traces from the initial training sessions on simple whisker (left) or odor (right) discrimination, 20-trial moving window (*N* = 5 and *N* = 6, respectively). Criterion is 80% correct responses in a 20-trial moving window. Rank sum comparison for trials to criterion across modality, *p* = 0.2. ***F***, Box plots of number of trials completed in the set-shifting task per session. *N* = 13 animals; 274 sessions; 123,482 trials; 1,108 trial blocks. ***G***, Box plots of trials to criterion for whisker and odor discrimination blocks during set-shifting for the same 13 animals from ***F***. Rank sum *p* = 0.1. ***H***, Accuracy (percentage trials correct) for trials aligned to the rule switch (mean ± SD across the same 13 animals from ***F***, ***G***). ***I***, Mean ± standard deviation, trial accuracy by modality rule, trial congruency, and trial timing relative to rule switch (within 10 trials pre- or postrule shift) for the sessions in ***F***–***H***. Three-way ANOVA performed on the 20-trial periswitch window yielded significant effects of congruency (*F* = 1,323; *p* ≈ 0), trial timing (i.e., whether a trial was within the last 10 trials prior to a shift or the first 10 trials following a shift; *F* = 1,752; *p* ≈ 0), and the interaction of congruency × timing (F = 5.11; *p* = 0.02), but not for modality or any interactions with modality. Horizontal red lines indicate Bonferroni-corrected significance for group differences.

**Figure 4.**
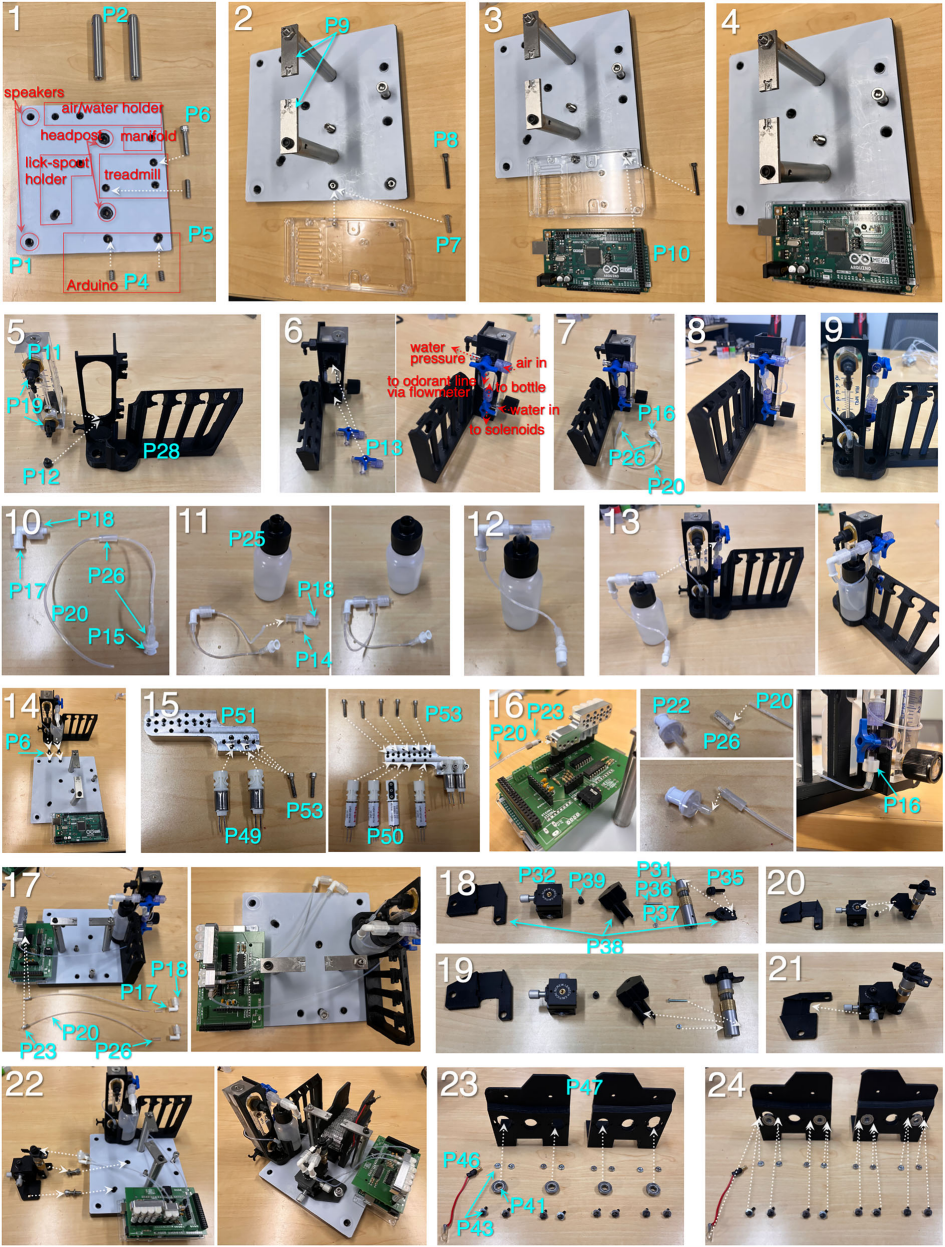
Assembly instructions (Steps 1 through 24). (1) Cover a 6″ × 6″ optical breadboard (Part P1; Table 1) with self-adhesive polytetrafluoroethylene (PTFE, P3) and cut through-holes for 1/4-20 screws. (2) Mount two 3″ × 1/2″ optical posts (P2). Insert adapters for mounting Arduino (P4) and set and cap screw for treadmill base. (3) Fasten Arduino case using a 4-40 button-head screw. (4) Fasten Arduino using a 4-40 cap screw. (5) Fasten flowmeter (P11) to air/water holder (P27) via an M5 cap screw (P12). Fasten NPT elbow barbs (P19) to flowmeter. (6) Attach Luer three-way stopcocks (P13) to an air/water holder. (7) Assemble connection from air intake stopcock to flowmeter by inserting a 6″ length of 1/16″ O.D. Teflon tubing (P20) into a 1/2″ length of 1/32″ I.D. silicon tubing (P26) at each end. At the stopcock end, couple the tubing to a 3/32″ male Luer barb (P16). The Teflon tubing should go inside the barb, silicon tubing outside the barb. (8) Thread male Luer barb onto a bottom female port on the air intake stopcock. (9) Couple Teflon/silicon tubing assemble to the barb on the flowmeter intake port. (10) Assemble connection from water intake stopcock to water bottle by inserting an 8″ length of 1/16″ O.D. Teflon tubing (P20) into a 1/2″ length of 1/32″ I.D. silicon tubing (P26) at each end. Move the silicon segment on the bottle side of the assembly to the middle of the assembly. Couple the stopcock side of the assembly to a 3/32″ female Luer barb (P15). (11) Insert the bottle end of the Teflon tubing through a Luer T-splitter (F/F/M, P14) at a right angle, so that the tubing exits the splitter through the bottom port. Attach a Luer male thread piece (P18) to the male port of the T-splitter. (12) Insert the free end of the Teflon tubing from the assembly into a 2 oz Luer-coupled water bottle (P25) and couple the bottle to the T-splitter. (13) Slot the water bottle onto the air/water holder and couple the T-splitter assembly from the water bottle to the air intake stopcock. (14) Fasten the air/water holder assembly to the base using low-profile 1/4-20 cap screws (P6). (15) Attach a pair of two-way 12 V solenoid valves to the solenoid face mount (P51) using three 2-56 cap screws, 1/2″ in length (P53). Note: the use of face-mount solenoids here, which requires a custom-machine face mount (P51), is optional. This design was favored as it enables the use of threaded ferrules for coupling the water and air tubing, which provides stability and security for these connections. Free-standing solenoids, which couple directly to tubing, are available from the same manufacturer. Attach a set of four three-way 12 V solenoid valves to the solenoid face mount. (16) Couple the water intake port on the solenoid face mount (side port) to the water intake stopcock. Create a tubing assembly by coupling a 12″ length of Teflon tubing (P20) to a 6-40 threaded ferrule (P23) on one end and a 3/32″ male Luer barb (P16) on the other end. (17) Create tubing assemblies for left and right lickspouts by coupling 2 8″ lengths of Teflon tubing (P20) to 6-40 threaded ferrules (P23) on the one end and F/M Luer elbow connectors (P17) on the other. Screw the ferrules from these assemblies into the left and right water output ports on the front of the solenoid face mount. (18) Attach the lickspout holder (P38) to the vertical translation post (P31) using a plastic 8-32 thumb screw (P35). Note: the use of plastic here is essential, to prevent current conduction between the left and right lickspouts. (19) Attach the vertical translation post to the post holder piece using a 3/4″-long 2-56 screw and nut. (20) Attach the post holder to the XY translation stage (P32) using a 1/4″-long 8-32 cap screw (P39). (21) Slide the XY translation stage to the base of the lickspout holder. (22) Attach the lickspout holder assemble to the base using 1/4-20 cap screws. (23) Assemble the treadmill by first inserting the steel axle bearings (P41) into the front and rear bearing slots on the treadmill holder base. (24) Secure the bearings to the treadmill holder base using 5/16″-long 4-40 screws and nuts (P43).

**Figure 5.**
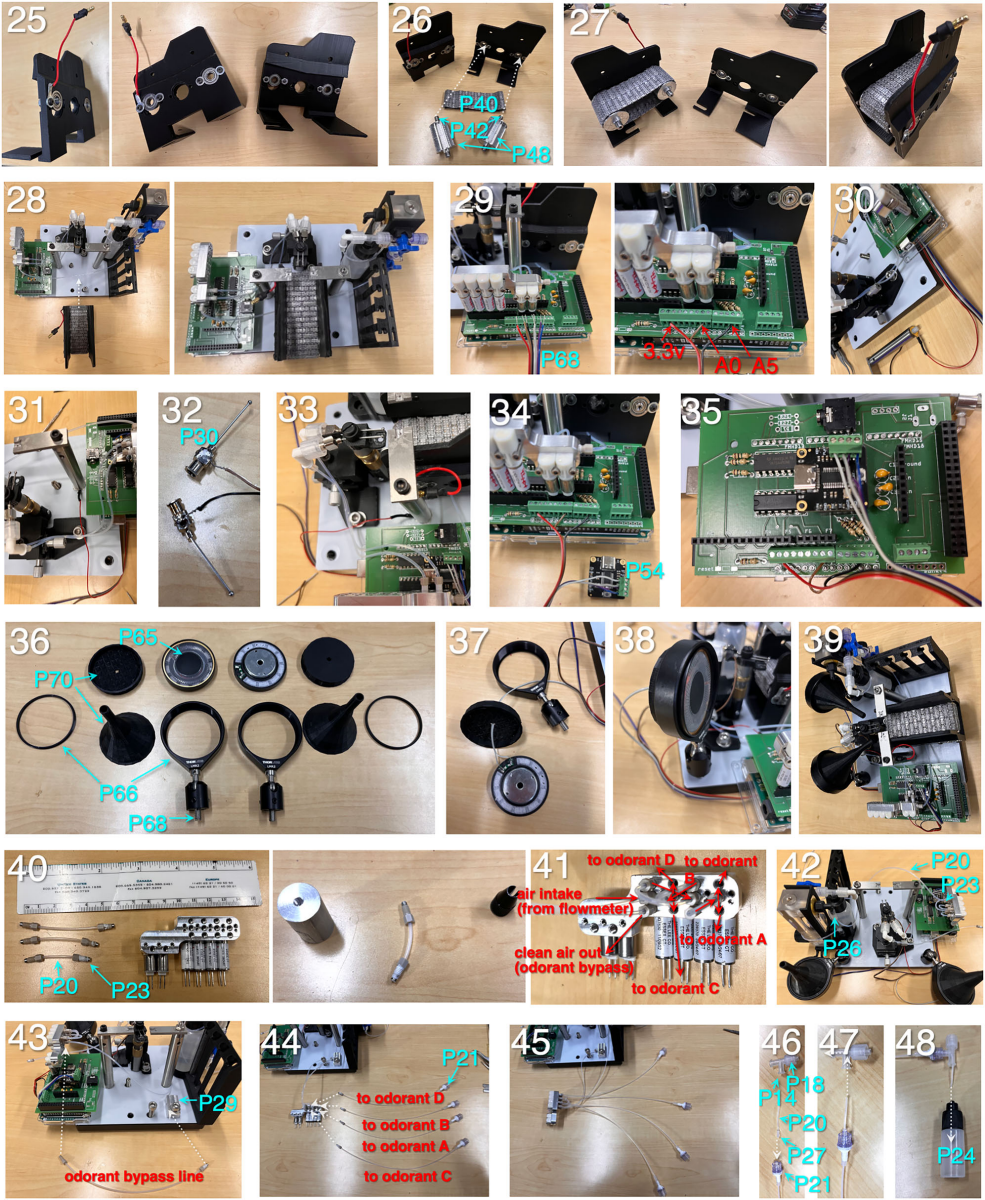
Assembly instructions (Steps 25 through 48). (25) To the left front bearing screw, couple a 2″ length of wire and a 3 mm banana plug (P46). (26) Insert 2″ posts (P42) into treadmill sprockets (P48) to serve as axles. (27) Insert sprocket axles into front and rear bearings, and mount treads (P40) around front and rear sprockets. 28) Slot the completed treadmill assembly onto the base using the set screw and cap screw that protrude from the base. Adjust the height of the rear cap screw so that the treadmill base slots securely beneath it. (29) Separate and twist the ends of a seven-wire ribbon cable (P68), and insert three of the wires into the screw terminals corresponding with the 3.3 V, A0, and A5 channels on the PCB board. (30) Couple the other end of the ribbon cable (∼10″ in length) to the base of the left head post using a brass lug or bare wire. (31) Screw the left head post back onto the base. (32) Solder the wires corresponding with channels A0 and A5 to the left and right lickspout gavage needles (P30), respectively. Secure gavage needles to the lickpost holder using the plastic 8-32 thumb screw. (33) Insert the banana plug into the side hole near the top of the left head post. Current of 3.3 V will now flow as follows: Arduino→ribbon wire→head post→banana plug→4-40 cap screw→steel bearing→axle and sprocket→treadmill→animal→gavage needle→ribbon wire→Arduino analog input channels. (34) Connect the four remaining wires from the ribbon wire bundle to the left +/− and right +/− screw terminals of the sound board (P54). (35) Insert the header pins from the sound board to the corresponding sockets on the PCB board. The sound board can be loaded with .wav files by connecting it to a computer via USB connection. (36) Assemble speaker unit pieces. (37) Thread left +/− and right +/− wires from the ribbon wire bundle through the ball-and-socket swivel mount (P66) and speaker backing piece, then soldering to the +/− contacts on the speaker (P65). (38) Set the speaker backing piece into the swivel mount, followed by the speaker. Fasten the swivel mount to the base by screwing an 8-32 to 1/4-20 thread adapter (P68) into the bottom of the swivel mount. Loosen the ball to allow it to rotate *ad libitum*, then screw it to the base, and re-tighten. (39) Fasten the speaker funnel (P70) to the front of the speaker assemble using a retaining ring. (40) Create three tubing-ferrule assemblies, using 2.5″ of 1/16″ O.D. Teflon tubing for one and 2″ of the same tubing for the other two assemblies. Couple to 6-40 threaded ferrules by feeding the tubing through the ferrule so that it is flush with the ferrule tip, then screwing tightly into a 6-40 threaded socket. Wrap ferrule threads in a Teflon tape to ensure tight seals. (41) Connect the tubing assemblies from Step 40 to the solenoid face mount. For the four three-way solenoids used to direct air flow for odorant delivery, the middle port on each solenoid is the intake. With the solenoid is in an “off” state (0 V, the bottom port is the exhaust, and with 12 V applied across the solenoid pins, the top port is the exhaust. The leftmost 3-way solenoid (when viewed from the tubing side of the face mount) directs clean air directly to the animal when off, and toward the middle/intake port of solenoid 3 (third from left) when activated. Solenoid 3 routes air to odorants A and B when off and to odorants C and D when activated. Solenoid 2 routes air to odorant C when off and D when activated. Solenoid 4 routes air to odorant A when off and to odorant B when activated. (42) Create a tubing assembly to direct air from the flowmeter to the odorant solenoids. Use a 10″ length of Teflon tubing (P20). On the flowmeter/intake side, couple the tubing to the top/exhaust port of the flowmeter using a 1/2″ length of 1/32″ I.D. silicon tubing (P26). Couple the exhaust side to a 6-40 threaded ferrule (P23) and screw into the solenoid face mount. (43) Create an odorant bypass line using an 8″ length of Teflon tubing (P20), coupled to 6-40 threaded ferrules at both ends (P23). Screw one end of the assembly into the odorant bypass port (bottom port of the leftmost solenoid), and screw the other end into the odorant manifold (P29). Note: the use of a custom-milled aluminum manifold here was adopted in order to keep a small footprint on the rig and to ensure a secure connection. Those wishing to avoid the need for this custom piece can substitute a barbed or Luer-style manifold. (44) Create four odorant intake tubes to connect the odorant solenoids to the odorant bottles. For each, use an 8″ length of Teflon tubing (P20), coupled to a 6-40 threaded ferrule on the one side (P23) and a barbed Luer check valve on the other (P21). The use of check valves on both sides of each odorant tube prevents backdrifting and comingling of odorants. (45) Screw the tubing assemblies into the solenoid face mount according to the mapping shown in Step 41. (46) To route the odorant intake air to the odorant within the bottle, insert a 1–1.5″ length of Teflon tubing (P20) into a 1/4″ length of 1/8″ O.D. 1/16″ I.D. (P27) silicon tubing. (47) Mount this tubing assembly into the tip of the male intake Luer check valve, and guide the tip of the Teflon tubing through the Luer T-splitter at a right angle, so that it protrudes from the other female Luer port. (48) Insert the protruding Teflon tubing into the opening of a Luer-threaded 0.25 oz bottle (P24), and screw the Luer connector securely.

**Figure 6.**
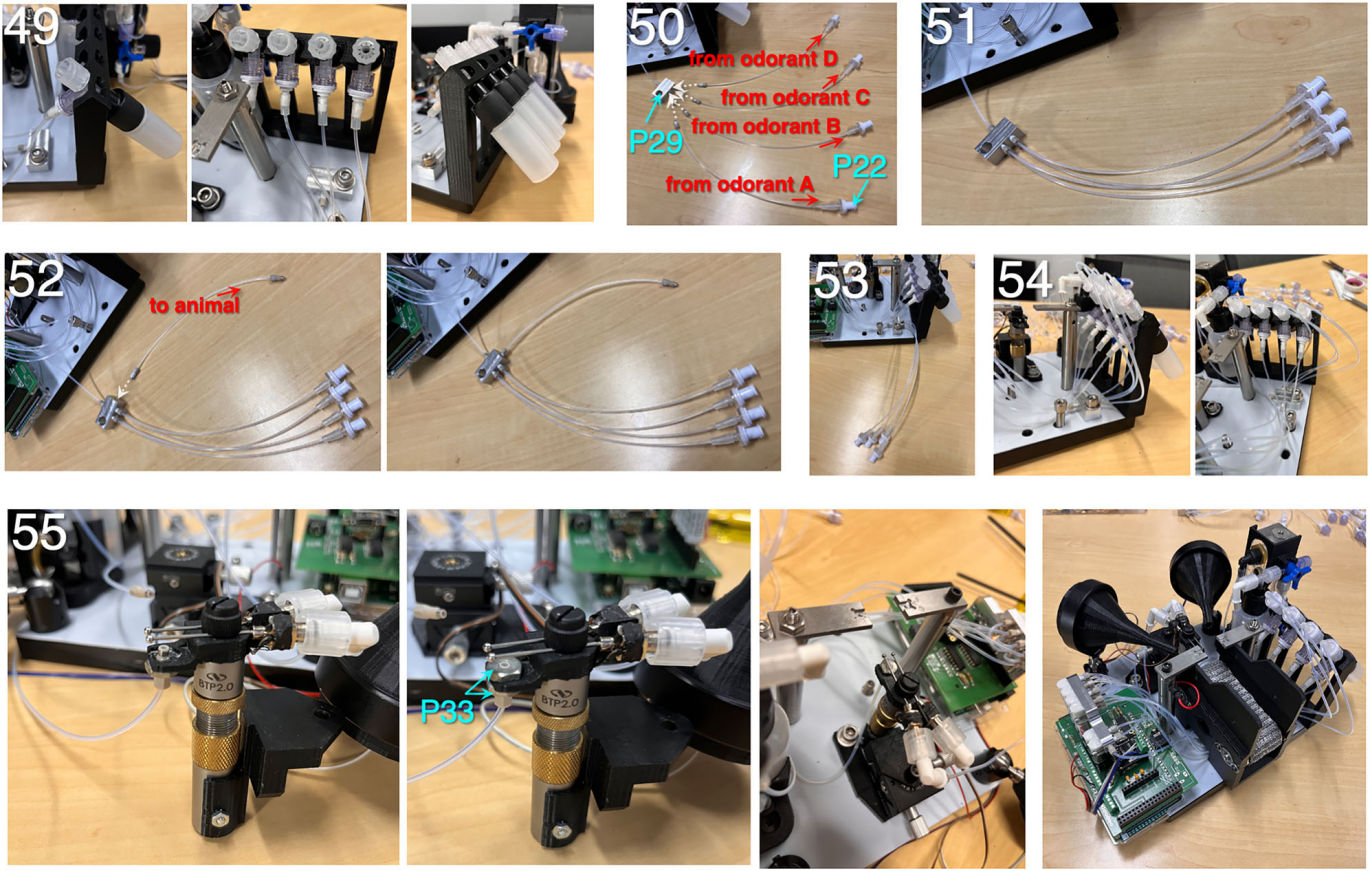
Assembly instructions (Steps 49 through 55). (49) Snap the T-splitters into the corresponding grooves on the odorant bottle holder unit. Repeat for all four odorant bottles. (50) Create a set of four odorant exhaust tubing assemblies, using 6″ lengths of Teflon tubing (P20) coupled to 6-40 threaded ferrules at one end (P23) and 3/32″ barbed Luer check valves at the other end (P22). (51) Screw the tubing assemblies into the odorant manifold (P29). (52) Create a 6″ tubing assembly to route air from the odorant manifold to the animal. Use a 6″ length of Teflon tubing (P20) coupled to 6-40 threaded ferrules (P23) at either end. Screw into the front port of the manifold. (53) Fasten the odorant manifold to the base using a 1/4-20 cap screw. (54) Attach the four odorant exhaust lines to the Luer T-splitters. (55) Attach the odorant output line to the lickspout holder using a pair of 6-40 nuts (P33).

FlexRig is an open-source, modular behavioral platform to facilitate replicability and collaboration (Figs. 1–3). While it was specifically designed to administer an olfactory/somatosensory cross-modal attentional set-shifting task, the system can be used to administer a wide range of behavioral tests involving whisker vibration, auditory, and/or olfactory stimuli, liquid rewards, and licking behavior. It consists of (1) hardware, in the form of a parts list of commercially available components (Table 1), (2) a set of 3D CAD files for the printing and machining of custom components, (3) a printable PCB circuit board design, (4) a set of assembly instructions (Figs. 4–6), and (5) a library of Arduino code (component functions and example task scripts) to run the platform.

**Table 1.** A table of rig components and commercial sources

Part number	Component	Item description	Number	Usage notes	Part ID	Supplier
P1	Base + head restraint	6″ × 6″ breadboard	1	Platform for all components	MB6	Thorlabs
P2		3″ × 1/2″ posts	2	Supports head plates	TR3	Thorlabs
P3		Adhesive Teflon sheet, (PTFE) 12″ × 12″	1	Covers the base, makes for easier cleanup	3M	3M
P4		Thread adapter, internal 4-40, external 1/4″-20	2	Mounting Arduino to the base	AE4E25E	Thorlabs
P5		1/4″-20 set screw, length 0.5″	1	Slotting treadmill front	91375A537	McMaster-Carr
P6		1/4″-20 cap screw, length 0.5-1″	3	Slotting treadmill, rear, fastening air/water holder to the base	92220A183	McMaster-Carr
P7		Button-head screw	1	Hold the Arduino case to the base	92949A110	McMaster-Carr
P8		4-40 cap screw, 1″ length	1	Hold Arduino to the case and base	91251A110	McMaster-Carr
P9		Head bars	2	Fastens head plate to head posts	headbar.stl	Printer
P10		Arduino Mega 2560	1	Control components and log behavior data	1,050-1,018-ND	DigiKey
P11	Air/water control	Flowmeter, 50 mm; 0.5 LPM Air	1	Regulates airflow to odorants	2968K201	McMaster-Carr
P12		M5 screw	1	Secures flow meter to holder	92290A277	McMaster-Carr
P13		Luer three-way stopcock, F/F/M	2	Air and water intake	PVAP-MFF-PC	ISM
P14		Luer three-way T-splitter, F/F/M	5	Coupled to water and odorant bottles	CFFT-WN	ISM
P15		Luer 3/32″ barbed connector, F	1	Connects water bottle to water intake stopcock	CFLLA-332-N	ISM
P16		Luer 3/32″ barbed connector, M	2	(1) Air intake to flowmeter; (2) water intake to solenoids	CIML-332EA-WN	ISM
P17		Luer elbow adaptor, M-F	3	(1) Water bottle to intake; (2–3) lickspout needles	CFLET-WN	ISM
P18		Luer snap-on threads	8	Secure male Luer adapters to female Luer adapters	CLFSR-WN	ISM
P19		1/8 NPT fitting (3/32″ barb)	2	Connecting 1/16″ PTFE tubing to flowmeter	F325483	ISM
P20		PTFE (Teflon) tubing, 1/16″ OD, 1/32″ ID	35'	Air and water lines	PTFE-132116-100	ISM
P21		Luer 3/32″ barbed check valve, intake	4	Air intake to the odorant tube	BCV-67220-ML	ISM
P22		Luer 3/32″ barbed check valve, exhaust	4	Air exhaust from the odorant tube	BCV-67FL-220	ISM
P23		6-40 threaded ferrules	24	Coupling to for 1/16″ OD tubing	SKU 640FF-16	Global FIA
P24		1/4 oz Luer-capped dispensing bottles	4	Contains odorants	JG0.25BC	Jensen Global
P25		2 oz Luer-capped dispensing bottle	1	Contains water	JG2.0BC Bl2	Jensen Global
P26		Silicon tubing, 1/32″ I.D., 5/32″ O.D.	10″	Coupling 1/16″ PTFE tubing to barbs	57286	US Plastics
P27		Silicon tubing, 1/16″ I.D., 1/8″ O.D.	10″	Coupling 1/16″ PTFE tubing to male Luer connectors at odorant bottles	57287	US Plastics
P28		Air/water holder	1	Holds flowmeter, water bottle, odorant bottles, and Luer connectors	airWaterHolder.stl	Printer
P29		Odorant manifold	1	Merges odorant exhaust channels	threadedManifold.emsx	CNC mill
P30	Lick stage	Gavage needle, 20 g	2		AFN2025S	GavageNeedle
P31		Translating post	1	Hold lick ports	BTP2.0	Newport
P32		XY translating stage	1	Hold lick ports	M-MT-XY	Newport
P33		6-40 nuts	2	Hold odorant tube	94812A663	McMaster-Carr
P34		6-40 threaded ferrules	18	Coupling to for 1/16″ OD tubing	SKU 640FF-16	Global FIA
P35		Plastic 8/32″ thumb screw	1	Securing lickspouts to holder	94320A393	McMaster-Carr
P36		2–56″ × 5/8″ screw	1	Securing translating post to holder	91802A083	McMaster-Carr
P37		2–56 nut	1	Securing translating post to holder	90480A003	McMaster-Carr
P38		Lickspout holder	1	Securing lickspouts to the XY stage and base	lickSpoutPostHolder.stl	Printer
P39		8–32 cap screw, 1/4″ length	3	(1) Lickspout holder to the XY stage, (2) head bars to posts	90128A191	McMaster-Carr
P40	Treadmill	Toy tank treads	76 links	Supports mouse, conducts lick detection current	TT35056	Warp United
P41		Steel bearings	4	Treadmill axle bearings	7804K143	McMaster-Carr
P42		2″ mini posts	2	Treadmill axles	MS2R	Thorlabs
P43		4–40 screws and washers	1 kit	Securing bearings to treadmill frame	ALAST0440	Fastener Express
P44		Rotary encoder	1	Measure running speed	AMT103-V	DigiKey
P45		1/4-20 cap screws, 1/2″	4	Secure treadmill to breadboard	92220A183	McMaster-Carr
P46		3 mm banana plug	1	3.3 V lick detection voltage connector	generic	Amazon
P47		Mouse platform	1	Supports mouse/treadmill	treadmillBase.stl	Printer
P48		Treadmill sprockets (aluminum)	2	Holds treads	treadmillSprockets.stl	Printer
P49	Command stage	Two-way solenoid valves, 12 V, face-mounted	2	Solenoid valves for dispensing water reward	LHDB1252115H	The Lee Company
P50		Three-way solenoid valves, 12 V, face-mounted	4	Route air to one of the four odorant tubes or bypass	LHDA1221411H	The Lee Company
P51		Air/water face-mount manifold (aluminum)	1	Mount solenoids to tubing	threadedFaceMount.emsx	CNC mill (e.g., Hubs)
P53		2–56 solenoid retention screws, 0.5″ length	8	Fasten solenoids to face mount	91251A081	McMaster-Carr
P54		DF Robot DF Player Pro	1	Play auditory and whisker somatosensory stimuli	1738-DFR0768-ND	DigiKey
P55		H-bridge	2	Gate 12 V power to solenoids and actuators	296-9911-5-ND	DigiKey
P56		six-pin screw terminal, 2.54 mm pitch	2	Connecting wires to PCB board	A98337-ND	DigiKey
P57		two-pin screw terminal, 2.54 mm pitch	1	Connecting linear actuator to PCB board	A98333-ND	DigiKey
P58		Eight-pin male header	6	Connecting PCB board to Arduino	732-5321-ND	DigiKey
P59		two-pin female header	6	Connecting solenoids to board	2057-RS1-02-G-ND	DigiKey
P60		six-pin female header	2	Connecting sound board to main board	2057-RS1-06-G-ND	DigiKey
P61		2 × 18 pin stackable female header	1	Connecting PCB board to Arduino	SSW-118-03-G-D-ND	DigiKey
P62		1 MΩ resistors	11	Dropping current (pull-down)	CF14JT1M00TR-ND	DigiKey
P63		Ribbon cable	1'	Connection from PCB board to components	3M157841-1-ND	DigiKey
P64		PCB board	1	Connects Arduino to peripheral components	FlexRigShield.fzz	Printer
P65	Vibration stage	Speakers (Tymphany, 20 Hz–20 kHz)	2	Auditory and whisker stimuli	HPD-50N25PR00-32-ND	DigiKey
P66		Locking Ball and Socket Mount	2	Mounting speakers to platform	TRB1	Thorlabs
P67		Lens Mount with Retaining Ring for Ø2″ Optics	2	Mounting speakers to platform	LMR2	Thorlabs
P68		Adapter 8–32 Threads and 1/4-20 Threads	2	Mounting speakers to platform	AP8E25E	Thorlabs
P69		Two-pin screw terminal, 2.54 mm pitch	2	Connecting linear actuator to PCB board	PRT-10571	Sparkfun.com
P70		Speaker funnels	2	Condensing sound wave, directing toward whiskers	speakerfunnel.stl	Printer

The left column (Part number) corresponds with blue labels in Figures 3–6, assembly instructions. Online product links and recent prices (accurate as of manuscript submission date) are available in spreadsheet format at the FlexRig Github page (spellmanlab/FlexRig).

The hardware modules include the following:

1. A base for securing a head-restrained mouse under a microscope or electrophysiology rig

2. A water module for delivering water rewards

3. A lick detection module for translating left/right licks into a behavioral readout

4. An olfactory presentation module to enable the delivery of up to four odorants in a given experiment

5. An air vibration module for administering auditory (∼5–20 KHz stimuli) and/or whisker somatosensory (20–300 Hz stimuli) cues

6. A command stage for interfacing the rig hardware components with an Arduino control board

What follows is a brief description of each module, as well as general instructions for assembly. Illustrated step-by-step instructions are included in the online project repository.

10.1523/ENEURO.0364-24.2024.d1 FlexRig Repository Download FlexRig Repository, ZIP file.

### Base

Conscious of space constraints that limit behavioral throughput in most labs, we designed the system to sit on a 6″ × 6″ aluminum optical breadboard with a 6″ × 6″ grid of 1/4-20 imperial threaded holes (Fig. 1). To facilitate ease of cleaning, the base is masked with a sheet of self-adhering PTFE. A pair of steel optical posts (1/2″ diameter, 3″ length) supports a pair of CNC-milled steel head bars (headbar.stl). These head bars, in turn, hold the mouse's surgically affixed head plate (headplate.stl, CNC-milled or 3D printed in aluminum or steel) via 0-80 screws.

### Water module

Water is dispensed under constant air pressure (optimally 3 psi) from a 2 oz water bottle with a Luer-locking adapter cap (Jensen Global). Water is pushed from the bottle through 1/16″ outer diameter PTFE tubing, which clears the bottle opening with sufficient space between the tubing and bottle aperture to permit the intake air to pressurize the bottle. This approach, of using the space around the tubing for air intake and the tubing itself for outflow, is also used to control airflow through the odorant bottles in the olfactory stimulation module. The water bottle and its associated Luer-locking intake/outflow components are held to the base by a 3D-printed piece (airWaterHolder.stl). Water flows from the water bottle to a pair of two-way solenoids (The Lee Company) on the command stage, and from the solenoids, water flows to a pair of Luer-locking steel gavage needles, which also serve as lick detectors (see below, Lick detection module).

### Lick detection module

Lick detection is achieved by completing a 3 μA circuit through the animal. Arduino's 3.3 V channel is connected, via a 1 MΩ resistor, to the animal via a tin alloy treadmill. We chose a treadmill, rather than a fixed platform, because prolonged head restraint can induce stress, which can be reduced through stationary locomotion (Juczewski et al., 2020), and animals using FlexRig exhibit spontaneous treadmill running, even in the absence of any explicit locomotor component in the task (Fig. 2*A*,*B*). The treadmill conducts the lick detection current between Arduino's 3.3 V channel and the lickspouts. More specifically, a wire connects the 3.3 V channel (via screw terminal on the PCB shield) to the left head post. From there, a banana plug conducts the current to a treadmill axle and bearing on the 3D-printed treadmill holder (treadmillBase.stl), which conducts the current through an aluminum sprocket (treadmillSprockets.stl, 3D-printed or CNC-machined), which contacts the treadmill belt that supports the animal. The treadmill base can be easily removed for cleaning by disconnecting the banana plug (3.3 V channel) from the head post and sliding the base backward. The steel gavage needles that dispense water to the animal are coupled to two of the Arduino's analog input channels, thereby serving as lick detectors. The water-dispensing gavage needles are precisely positioned relative to the animal by a 3D-printed piece (lickSpoutPostHolder.stl). A vertically translating lens mount (Newport) allows for fine dorsal–ventral adjustment of the lickspout positioning, while a miniature 1/4″-travel XY translating stage allows axial and lateral adjustment.

### Olfactory stimulation module

Air routed to the odor presentation system is branched from the same source (intake either from the building's air line or a compressed air tank) that provides constant pressure to the water module, via a three-way Luer stopcock. This air line is directed to an adjustable flow meter (0.3 Lpm is typically used). From this flow meter, air travels to a set of four three-way solenoid valves at the command stage. The first of these solenoids controls the air bypass, whereby clean air is channeled directly to the animal between odorant presentations via a manifold. When this first solenoid is activated, air is redirected to the three solenoids that control airflow to the four odorant bottles. These odorants are contained in 0.25 oz plastic bottles (e.g., eugenol, benzaledehyde, isoamyl acetate, and limonene, each diluted 1:100 in mineral oil, 1 ml in each bottle), which are held in place by a 3D-printed piece (airWaterHolder.stl). Both intake and outflow on the odorant bottles are controlled by check valves that prevent backward flow of odorant air into other bottles. From the odorant bottles, air channels converge at a CNC-milled manifold (mstacManifold.emsx—the clean bypass air channel converges here with the output lines from the odorant bottles). The outflow channel from the manifold terminates at the lickspout holder, where it is fastened beneath the animal's nose and directed vertically toward the nose. While we do not incorporate an active exhaust system to clear odorants from the nose area following trials (apart from a 5 V outward-facing exhaust fan at the rear of the isolation box), we observe rapid odorant clearing through diffusion, within 2 s of valve offset (Fig. 2*C*), and we do not detect accumulation of odorants over repeated trials. This is supported by the observation that mice perform 450 ± 50 trials per session, without a significant difference in discrimination performance between whisker and odor cues (Fig. 3*G*,*I*).

### Air vibration module

While various solutions to the challenge of inducing discriminable whisker stimulation have been used, including magnetic field manipulation (Melzer et al., 1985), rotation of textured cylinders (Lottem and Azouz, 2009), and linear actuator-based displacement (Galvez et al., 2009), we opted for vibration of the whiskers using low-frequency sound waves. Miniature broad-spectrum stereo speakers (Tymphany, 20 Hz–20 kHz) provide multiplexed auditory and patterned whisker vibration stimuli. These speakers were selected to satisfy two criteria: a small footprint (<2″ diameter) and performance at the ultralow frequencies needed to oscillate rodent whiskers, which have resonant frequencies ranging from 25 to 500 Hz (Hartmann et al., 2003; Neimark et al., 2003; Boubenec et al., 2012). A sound card with built-in preamplifier (DFRobot) is preloaded with .WAV files (see Tones Folder in Extended Data for example tones) via the USB port. The speakers are wired to screw terminals on the sound card and mounted onto the base with 2″ ball-and-socket-style lens mounts (Thorlabs). Sound waves are condensed and directed toward the mouse's whisker vibrissae and ears via 3D-printed funnels (speakerFunnel.stl) that fasten to the lens mounts via threaded retaining rings. Whisker vibration frequencies spanning 35–210 Hz produce displacement of vibrissae of varying lengths (Fig. 2*E*) that correspond with published values capable of inducing widespread and robust spiking in the trigeminal ganglion cells, >50% of which respond to whisker deflections of <1°(Gibson, 1962).

### Command stage

The command center for the water, lick, olfactory, and vibration modules is a custom-designed Arduino shield (FlexRigShield.fzz; Fig. 1*C*). Connection wires for 3.3 V lick signal, analog lick detection, speakers, as well as other command channels for additional inputs/outputs (e.g., optogenetic or other closed-loop command signal) couple to screw terminals on the shield, and solenoid valves for water and olfactory modules plug directly into the board, as does the sound card.

### Tubing termination and fastening

All air and water lines on the rig use tubing (PTFE “Teflon,” 1/16″ O.D., 1/32″ I.D.). Use of this material confers several advantages. First, PTFE resists the buildup of films and residues that can harbor mold over time and result in lingering odorants. Second, the narrow gauge of the tubing makes for a small footprint on the rig and results in rapid transmission of odorants from the odorant tubes to the animal (∼20 ms latency at a flow rate of 0.3 Lpm). Third, the rigidity of the material, relative to other tubing composites such as silicone, resists kinks, pinches, and breaks. This tubing is coupled in two ways in this rig design: first, it is coupled to barbed Luer lock connectors (3/32″; Fig. 4, assembly instructions, step 16). When coupling to these barbs, the rigid, narrow PTFE tubing is first pushed through a ∼1/2″ length of silicon tubing (1/32″ I.D., 3/32″ O.D.), which forms a tight connection around the PTFE tubing. The internal PTFE tubing is then inserted into the barb, while the external silicon tubing is stretched over the exterior of the barb. The resulting connection is highly secure and resistant to pinching and kinking. Second, the narrow PTFE tubing can be coupled to a manifold by crimping to a 2–56 threaded ferrule (Global FIA or The Lee Company), which results in an even more secure connection than a Luer barb. In the design presented here, this threaded ferrule coupling is used in two places: to couple air and water to the solenoids at the command stage and to couple odorant lines to the odorant manifold. We present CAD files for these couplers (threadedFaceMount.emsx and theadedManifold.emsx, respectively), but for experimenters wishing to avoid the cost of machining these parts, solenoids that couple directly to tubing can be used (The Lee Company), and Luer lock manifolds can be used in place of a custom-milled, threaded manifold.

### Arduino interface

The platform is controlled using an Arduino library (flexRigFunctions.h) and the Arduino integrated development environment (IDE). Visualization and logging of behavioral data and selection of session parameters (see example scripts in Extended Data) can be done using Arduino IDE's native serial monitor or using a third-party serial interface program (e.g., Putty for Windows). After copying the flexRigFunctions folder to the computer's Arduino library directory, each of the basic modules can be controlled by calling a set of custom functions from an Arduino script, of which several examples are provided as follows:

1. initializeBoard: establishes serial connections, assigns and formats hardware pins, and starts the sound card

2. dispense: dispenses water from the lickspouts

3. playTone: plays a sound file from the speakers

4. killTone: interrupts an ongoing sound file

5. lickDetect: detects contact between the 3.3 V on the treadmill and the two lickspouts

6. presentOdorant: presents one of up to four odorants contained in the odorant bottles. When not activated, clean air is continuously routed to the mouse's nose, bypassing the odorant bottles

7. killOdorants: interrupts an ongoing odorant presentation

8. photoStim: outputs trains of frequency- and duration-specific TTL pulses to drive optogenetic stimuli (via Arduino pin 38) from an external light source (not included). This function makes use of the native TimerOne and TimerThree Arduino libraries to enable configurable stimulus trains to run in the background without interrupting the ongoing task program.

#### Animal habituation and training, attentional set-shifting protocol

The Arduino code base provided includes example scripts for training animals on an attentional set-shifting task. The training protocol is divided into three overall stages, each with corresponding scripts. Habituation to the apparatus and head fixation is performed using the *habuation.ino* script. Behavioral shaping, which pairs water rewards with stimuli to induce licking behavior, is performed using the *shaping.ino* script. These two scripts are broadly applicable to any behavioral task that requires left/right licking in response to whisker or odor stimuli, e.g., discrimination tasks. After completion of these training stages, the *bimodal_2AFC.ino*, which stands for “bimodal 2-alternative forced-choice,” is used to train the animal on the remaining stages of the set-shifting protocol, which is outlined below.

***Training protocol***. Thirteen C57/B6 mice (7 males, 6 females) were tested. All animals are housed on a reverse light/dark cycle (12 h each, 9A.M.–9P.M.) in a temperature- and humidity-regulated facility (20–24°C, humidity 30–60%). Food is made available *ad libitum* and changed weekly. One week following surgery, animals are placed on a water restriction regime, receiving 1–1.5 ml per day, body weight >85% of the baseline. Following 3 d of water restriction, mice undergo manual habituation by an experimenter. The experimenter lifts the mouse from the home cage using a gentle scooping motion and holds the animal in the experimenter's cupped, gloved hand, which is elevated ∼8″ above the open home cage. As the mouse explores the hand, seeking a route to return to the home cage, the experimenter places their free hand between the mouse and the open cage, prompting the mouse to climb into it. The experimenter then uses a hand-over-hand, treadmill-like motion to keep the mouse ambulating toward the cage. After 5 min of exploration, the experimenter administers water to the mouse orally, in −5 μl droplets, using a syringe and 20 gauge steel gavage needle. After allowing the mouse to consume up to 1.5 ml of water, the hand-over-hand exploration process is repeated for another 5 min, after which the mouse is returned to the home cage. This manual habituation protocol is repeated for 3 d.

Following manual habituation, the animal is habituated to the FlexRig. The *habuation.ino* script is uploaded, and in a serial window, the *HB1/2* program is selected. This program arms the lickspouts in a pseudorandomized alternating pattern, so that a 4 μl droplet is administered in response to a lick from one of the two lickspouts, with a 0.5–1.5 s timeout following water deliveries. All water deliveries are reinforced by a simultaneous 500 ms white noise tone delivered through the corresponding left/right speaker. In *HB1*, the first stage of habituation, the experimenter places the animal on the treadmill, using a water-filled syringe and gavage needle to bait the animal toward the lickspouts, and manually (gently) returning the animal to the treadmill each time it climbs off. The session ends when the animal goes 2 min without consuming water, and the animal is considered to have passed this stage upon consuming 500 μl of water in a session.

In *HB2*, the second stage of habituation, *habituation.ino* is again uploaded to the Arduino, and the *HB1/2* program is again selected in the serial window. Here, though, the animal is fixed in the head restraints using 0-80 screws. Care must be taken to orient the lickspouts at a position relative to the animal's mouth, using the XY stage and vertically adjustable post on the lickspout holder assembly, to allow the tongue to reach both. Figure 3*B* illustrates an optimal such positioning for the head angle used in our surgeries. However, as the angle of the head relative to the headplate can affect the optimal positioning of the lickspout relative to the nose, care must be taken to fine-tune this distance based on the animal's lick success for each experiment. As with HB1, in HB2 the experimenter ends the session when the animal goes 2 min without consuming further water, and the animal is considered to have passed when it has consumed 500 μl in a session.

In *HB3*, the animal is placed in the treadmill and restrained, and *HB3* is selected in the serial window. In this stage, trials are blocked in incrementally increasing block lengths of up to 20 trials (20 left trials, 20 right trials, etc.). Criterion for passing this stage is consumption of 500 μl of water in a session.

In habituation stage *HB3B*, a brief (150–300 ms) whisker stimulus is presented after licking and before water delivery, to establish an association between the whisker stimulus and left/right water location.

After passing *HB3B*, the animal proceeds through two shaping stages, *SH4* and *SH5*, which are administered using the Arduino script *shaping.ino*. On each trail in *SH4*, a bilateral white noise tone (500 ms) signals the onset of the trail. A frequency-specific whisker vibration stimulus is then presented (left, 210 Hz; right, Poisson-distributed quasi-square-wave pulses). This stimulus is administered for 2,500 ms, during which time the animal may lick either lickspout, although these licks do not affect reward delivery. Following the end of the stimulus, a response window begins, during which, a lick to the correct side triggers immediate reward delivery. The animal may lick the incorrect side prior to licking the correct side, and reward is still delivered. A 3,000–4,500 ms intertrial interval (ITI) follows each reward delivery. Criterion for passing *SH4* is 500 μl of water consumed within a session. Beginning with SH4, and for all subsequent training steps, including the final set-shifting task, behavioral sessions terminate when the mouse fails to perform a lick response on 10 consecutive trials to indicate satiety. Fully trained animals performing the set-shifting task complete 450 ± 50 trials per session (Fig. 3*F*).

The trial sequence for *SH5* is similar to *SH4*, but in *SH5*, the first lick during the response epoch must be to the correct lickspout in order to trigger water delivery. Criterion for passing this stage is that the animal must reach an accuracy of 80% within a 100-trial moving window at some point during the session.

After passing *SH5*, the animal proceeds to *simple discrimination* (*SD*), which is administered using the script *bimodal_2AFC.ino*. The trial structure and whisker stimuli are identical for *SD* and *SH5*, except that in *SD*, as well as all subsequent task stages, an ITI of 8,000–10,000 ms is used, and the blocked, 20-trial left/right alternation pattern is replaced by either (1) trial-by-trial randomization of the left/right reward location or (2) pseudorandomization with correction for directional lick bias. Lick bias correction is an option set by the experimenter in the Arduino script used for *SD* and subsequent task stages, and it consists of weighting the likelihood of selecting a given reward location in inverse relation to the animal's frequency of selecting that location in recent trials. Using lick bias correction in this way prevents the animal from adopting a stereotyped response strategy and forces the animal to instead form stimulus–response–reward associations. However, because lick bias correction can skew the distributions of behavioral variables, it is recommended that this option be deselected following training and during physiological recordings. Criterion for passing *SD* is fourfold. The animal must (1) perform >100 trials, (2) reach 80% accuracy in a 30-trial moving window, (3) simultaneously surpass 50% accuracy on both left and right trials within a 10 trial moving window, and (4) make a response in >80% of trials. After passing *SD*, the animal proceeds to *compound discrimination* (*CD*), which differs from *SD* in that, on each trial, one of two possible olfactory distractor stimuli is presented concurrently with the whisker stimulus. Criterion for passing *CD* is identical to that for *SD*.

Following acquisition *CD*, according to established set-shifting protocols, an animal is then tested on three forms of cognitive flexibility, *intradimensional set-shifting* (*IDS*), *reversal* (*Rev*), and *extradimensional set-shifting* (*EDS*), and the trial structure for all these sessions is illustrated in Figure 3*C*. *IDS* consists of acquiring new stimulus exemplars within an established stimulus modality (e.g., associating a new pair of whisker vibration patterns with left and right reward locations). *Reversal* entails remapping the left–right associations of a known pair of stimuli, e.g., so that a whisker vibration pattern previously signaling a left reward instead signifies a right reward and vice versa. *EDS* involves learning to associate stimuli within a previously irrelevant modality, in this case olfactory stimuli, with reward locations. *EDS* has most often been found to be the most challenging form of cognitive flexibility, requiring the transformation of a meta-association between abstract stimulus categories and response strategy (Tait et al., 2014). All three of these tests can be administered using the *bimodal_2AFC.ino* script and FlexRig. Additionally, the script includes a *serial extradimensional set-shifting program* (*SEDS*), which allows for repeated set-shifts within a session, enabling repeated sampling within an animal (Biró et al., 2019). In SEDS sessions, rule shifts are triggered when the animal attains 80% accuracy within a 30-trial moving window.

## Results

We trained a cohort of 13 mice (seven males, six females) on the set-shifting protocol using FlexRig. We observed anticipatory licking behavior, beginning at the onset of the stimulus and taking on a characteristic 6–8 Hz lick pattern previously reported in other lick-based tasks with mice (Murakami, 1977; Fig. 3*D*). In a cohort of 13 mice, each of which performed 19 ± 4.4 set-shifting sessions, we observed between 169 and 866 trials per session (Fig. 3*F*). To analyze behavioral and neural responses to rule switches from the odor attention rule toward the whisker attention rule and vice versa, it is important to determine whether stimuli of the two sensory modalities are equally detectable in the given task configuration. To test this, we compared the number of trials required to reach criterion performance on the two modality rules and found no significant difference (Fig. 3*G*). We also separately trained a smaller cohort (on the set-shifting paradigm, with one group of mice learning odor discrimination first and the other group learning whisker discrimination first), and we found no difference in trials to criterion (*N* = 6 and 5 animals, respectively; Fig. 3*E*). We next looked for performance differences across modalities before and after rule switches, when task accuracy transiently drops as the animal briefly persists in applying the previous rule before changing its strategy (Fig. 3*E*). We used a three-way ANOVA to model accuracy according to sensory modality (odor vs whisker rule), shift-aligned time (whether a trial occurred within the 10 trials before or after a rule shift), and congruency (whether the stimuli from two modalities indicated the same reward location, i.e., whether the animal needed to use the currently active attention rule to execute the correct response). Trial time and congruency were both significantly associated with response accuracy (three-way ANOVA with pairwise interactions; *N* = 123,482 trials across 274 sessions from 13 animals; *p* ≈ 0 for trial time and congruency; and *p* = 0.02 for their interaction), with accuracy dropping following the rule change for both congruent and incongruent trials. However, there was no effect of modality (*p* > 0.05), nor did modality interact with either of the other variables, indicating that the animals’ application of the task rule, both before and after rule changes, was paralleled across the two modalities (Fig. 3*F*). Some dropoff is observed in congruent trial performance following rule shifts, as seen in Figure 3*H*, and we attribute this mainly to stereotyped responses that are often adopted by mice after abandoning the previously successful rule and prior to acquiring the new rule—most often this takes the form of direction bias.

## Discussion

We present here an open-source platform for performing repeatable behavioral tasks involving multimodal sensory stimuli, including cognitive flexibility experiments, in mice. FlexRig boasts a number of useful features that allow users to create and execute their own behavioral experiments and do so at scale. Its small footprint, low cost compared with commercially available alternatives, minimal human involvement, and customizable functions all permit researchers a wide variety of potential tasks for animals. FlexRig's customizability and potential for open-ended integration with other systems allow it to be used in complex in vivo experiments, such as performing two-photon imaging and optogenetics in tandem with behavior. The small spatial footprint of the rig enables ready integration with existing experimental systems, such as two-photon laser–scanning microscopy.

We show the success of FlexRig in one such task, the attentional set-shifting task. The training for this task involved nine distinct steps and thousands of trials over several weeks. The same physical apparatus was used for each of these training steps and required very little touch time. From a single cohort of five animals, we were able to collect data from over 11,000 trials and demonstrate that for this task, animals apply the task rule equivalently irrespective of sensory modality. In the future, we hope to continue to integrate the FlexRig system with additional data streams, such as pupillometry and running speed, to create a more complete picture of animal behavior during complex tasks.

As the disease burden of psychiatric and neurological disease grows, so too does the need to build predictive theories and identify plausible therapeutic targets for failures in cognitive flexibility. Our hope is that the use of FlexRig will allow researchers to better collaborate and have a shared framework and platform for cognitive flexibility behavioral experiments and give a better understanding of how the brain adapts to its ever-changing environment.

## Synthesis

Reviewing Editor: Mark Laubach, American University Decisions are customarily a result of the Reviewing Editor and the peer reviewers coming together and discussing their recommendations until a consensus is reached. When revisions are invited, a fact-based synthesis statement explaining their decision and outlining what is needed to prepare a revision will be listed below. The following reviewer(s) agreed to reveal their identity: Ali Mohebi.

Thank you for sending your study to eNeuro! It was reviewed by two experts and their reviews are given in full below. Please revise your paper to address all points that were raised.

Please also consider preparing a visual abstract (a simple figure that captures the essence of your method). This is useful to the journal in promoting your work on social media.

Reviewer 1

This study introduces a novel open-source head-fixed platform designed for training mice on complex cognitive tasks, that can be used for advanced high-precision electrophysiology and imaging techniques to investigate the brain dynamics associated with these tasks. Overall, the manuscript is well-written and shows potential for valuable contributions to the field. However, there are a few minor issues that need to be addressed before publication.

The principal concern pertains to the justification for the development of a new platform. The manuscript lacks a thorough discussion and comparison with existing similar tools. A brief review through Google Scholar revealed over ten relevant references that are not cited in the manuscript and they all have developed similar systems, more or less. While it is acknowledged that different research labs may have unique design specifications tailored to their specific experiments, it is crucial to address the potential proliferation of disparate tools. The risk here is that if each laboratory develops its own unique platform, it could complicate the comparison of results across different studies and undermine the purpose of having open-source tools that facilitate collaborative research.

In an ideal scenario, research labs would build upon existing platforms by developing add-on modules rather than creating entirely new systems from scratch. There are significant institutional efforts aimed at this approach, such as the International Brain Lab and the Allen Institute, which are designing standardized platforms for head-fixed mouse experiments that integrate neurophysiology and optogenetics. The envisioned model is similar to an app store, where core platforms are universally used, and researchers develop supplementary modules to enhance these platforms' capabilities. This approach would streamline tool development and encourage broader adoption and innovation.

In addition to this major concern, there are a few minor points that need to be addressed:

1. Alongside the .stl files provided, please include the raw design files (e.g., .STEP files). This will allow users to modify the designs as needed to fit their specific experimental requirements.

2. The manuscript should specify the exact location of the spouts relative to the animal's snout. Accurate placement is crucial-spouts that are too close may lead to obsessive licking, while those positioned too far away may demotivate the animals. Including a schematic or photograph of the spout placement would be highly beneficial.

Addressing these points will enhance the manuscript's clarity and usefulness, and contribute to a more standardized approach in the field.

Please consider citing similar efforts. A few examples:

1. Ozgur, A., Park, S. B., Flores, A. Y., Oijala, M., &Lur, G. (2023). A comprehensive, affordable, open-source hardware-software solution for flexible implementation of complex behaviors in head-fixed mice. Eneuro, 10(6).

2. Gordon-Fennell, Adam, Joumana M. Barbakh, MacKenzie T. Utley, Shreya Singh, Paula Bazzino, Raajaram Gowrishankar, Michael R. Bruchas, Mitchell F. Roitman, and Garret D. Stuber. "An open-source platform for head-fixed operant and consummatory behavior." Elife 12 (2023): e86183.

3. Weaver, Isaac A., S. Aryana Yousefzadeh, and Michael R. Tadross. "An open-source head-fixation and implant-protection system for mice." HardwareX 13 (2023): e00391.

4. Cano-Ferrer, Xavier, Alexandra Tran-Van-Minh, and Ede Rancz. "RPM: An open-source Rotation Platform for open-and closed-loop vestibular stimulation in head-fixed Mice." Journal of Neuroscience Methods 401 (2024): 110002.

5. Groblewski, P. A., D. Sullivan, J. Lecoq, S. E. J. de Vries, S. Caldejon, Q. L'Heureux, T. Keenan et al. "A standardized head-fixation system for performing large-scale, in vivo physiological recordings in mice." Journal of Neuroscience Methods 346 (2020): 108922.

6. Hegedüs, Panna, Anna Velencei, Claire-Hélène de Belval, Julia Heckenast, and Balázs Hangya. "Training protocol for probabilistic Pavlovian conditioning in mice using an open-source head-fixed setup." STAR protocols 2, no. 3 (2021): 100795.

7. Lacefield, Clay, Hongtao Cai, Huong Ho, Carla Dias, Hannah Chung, René Hen, and Gergely F. Turi. "An Open-Source Virtual-reality System for Measurement of Spatial Learning in Head-restrained Mice." Journal of visualized experiments: JoVE 193 (2023).

8. Hughes, R. N., Bakhurin, K. I., Barter, J. W., Zhang, J., &Yin, H. H. (2020). A head-fixation system for continuous monitoring of force generated during behavior. Frontiers in integrative neuroscience, 14, 11.

9. Micallef, Andrew H., Naoya Takahashi, Matthew E. Larkum, and Lucy M. Palmer. "A reward-based behavioral platform to measure neural activity during head-fixed behavior." Frontiers in cellular neuroscience 11 (2017): 156.

10. Han, Zhe, Xiaoxing Zhang, Jia Zhu, Yulei Chen, and Chengyu T. Li. "High-throughput automatic training system for odor-based learned behaviors in head-fixed mice." Frontiers in neural circuits 12 (2018): 15.

11. Guo, Zengcai V., S. Andrew Hires, Nuo Li, Daniel H. O'Connor, Takaki Komiyama, Eran Ophir, Daniel Huber et al. "Procedures for behavioral experiments in head-fixed mice." PloS one 9, no. 2 (2014): e88678.

Reviewer 2

In this study, the authors present an apparatus for behavioral training of head-fixed mice that allows for multimodal stimulation, e.g. for attentional set shifting tasks, is easy to build and has a small footprint. The authors provide detailed instructions for the assembly of the rig, the software to operate it and a protocol on shaping and training mice with it. Further, they demonstrate in a cohort of mice that animals perform >300 trials in a training session, have comparable learning speeds for olfactory and whisker stimuli and show classic features of extradimensional set shifts, like an effect of congruency on the performance.

Overall, this study is a valuable resource for behavioral and systems neuroscience, fields in which learning paradigms in mice provide crucial insights. The instructions for building the setup are very detailed, containing a parts list, photos and supporting files for machined or 3D printed parts. Together, the setup seems easy to reproduce and thanks to the provided training software will likely create standardized, comparable data; an important goal for the neuroscience community. While I believe that the methods descriptions are nicely comprehensive, the results section giving a training example would benefit from additional clarification on how the protocols were applied in several (minor) points. I have listed suggestions below. Additionally, the olfactory stimulation protocol should be supported by calibration information (similar to what is provided for the whisker stimulation).

Suggestions:

In the Methods section, it would be good to see the odor delivery latency and, more importantly, the decay dynamics (since there is no active odor removal) measured with a PID. Especially, if the ultimate aim is to combine this setup with neural recordings, detailed information on the sensory stimulation is crucial.

The habituation and shaping protocols are laid out in a nicely detailed way. However, please don't lift mice by the tail and/or encourage other labs to do so by writing this in the protocol. There are several more refined methods that have long lasting effects on reducing their stress level without increasing experimenter efforts, for example tunnel handling or cupping. Here are two resources for more information: (https://www.nature.com/articles/s41598-020-71476-y, https://3rc.org/refined-mouse-handling/)

Regarding the Results section:

It would be good to include a schematic of the trial structure of the final task stage (stimulus, response window and ITI durations, reward delivery,..)

Similarly, more information on the session structure is needed: How long is a training session? When does it time out? As in, why do animals do different amounts of trials? What triggered a rule switch? How many were there in one session/per animal?

Regarding the learning speed, in the figure legend and main text the information of which statistical test was used is missing. Were mice only pretrained on whisker stimulation (as the protocol lays out) or on both modalities separately?

Which trials were taken into account for the ANOVA comparison? All trials in a session or only the -10 to 10 trials surrounding a rule switch? If really that many rule-switches happened then that is a very strong point that could be made more explicit in text.

It would be great to see a sliding window accuracy (or even single trial decisions) for one example session with rule-switches to get a better intuition for Fig 3F.

Is the animal moving on the treadmill or sitting still? A comment on this in text would be good.

Can you provide an interpretation why there is a performance drop for congruent trials after a rule switch?
