# Supplementary figures and images for: An Open-Source 3D-Printable Platform for Testing Head-Fixed Cognitive Flexibility in Rodents
Source: eNeuro. 2025 Jan 24;12(1):ENEURO.0364-24.2024. doi: 10.1523/ENEURO.0364-24.2024 (PMC11875048; doi:10.1523/ENEURO.0364-24.2024)

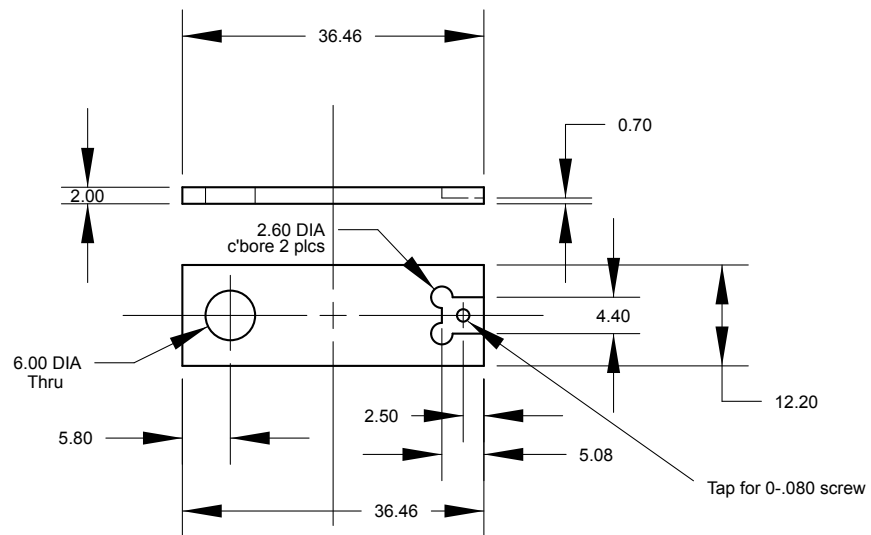

|        |            |                     |              |
|--------|------------|---------------------|--------------|
| F Moda | 09/08/2015 |                     |              |
|        |            | Plate               |              |
|        |            | Mat'l<br>Tool Steel | Sheet 1 of 1 |

Supplement: FlexRig Repository — Download FlexRig Repository, ZIP file. [file eneuro-12-ENEURO.0364-24.2024-s003.zip › FlexRig-main/3DModels/headbar.pdf]

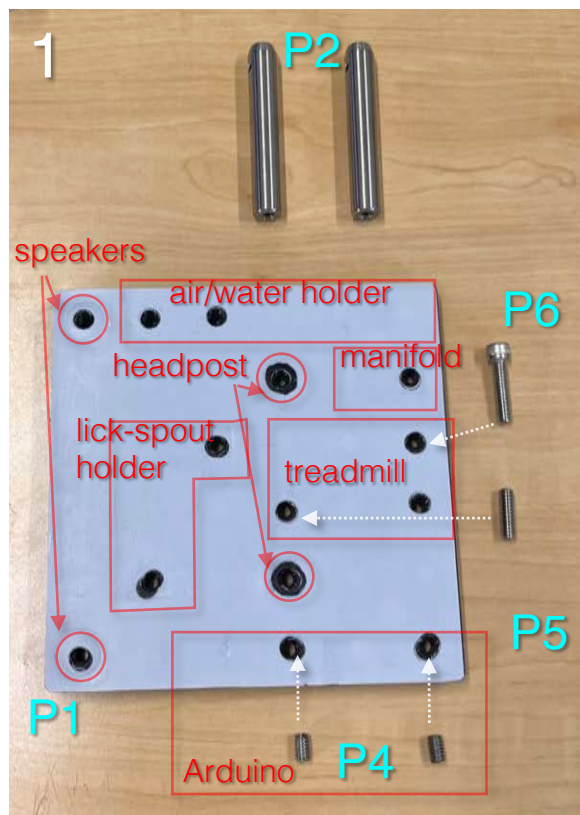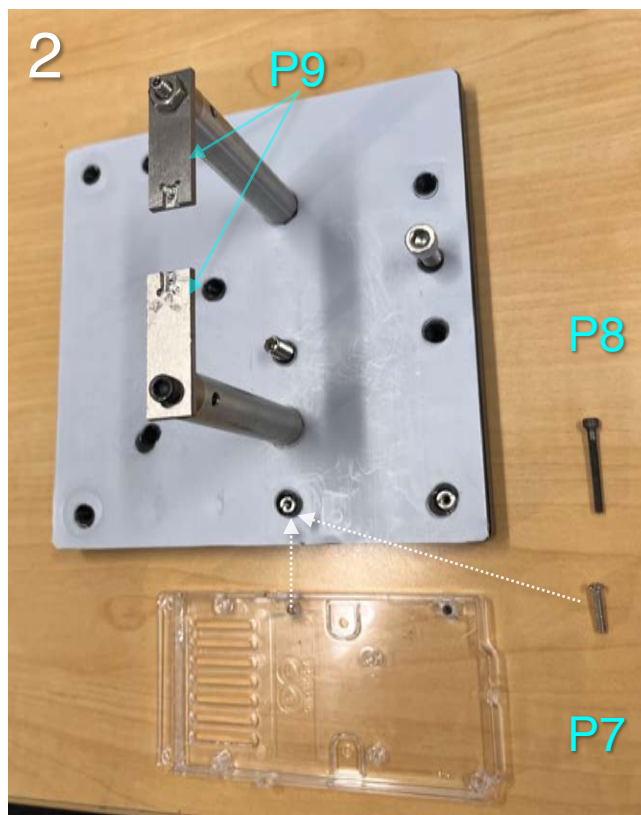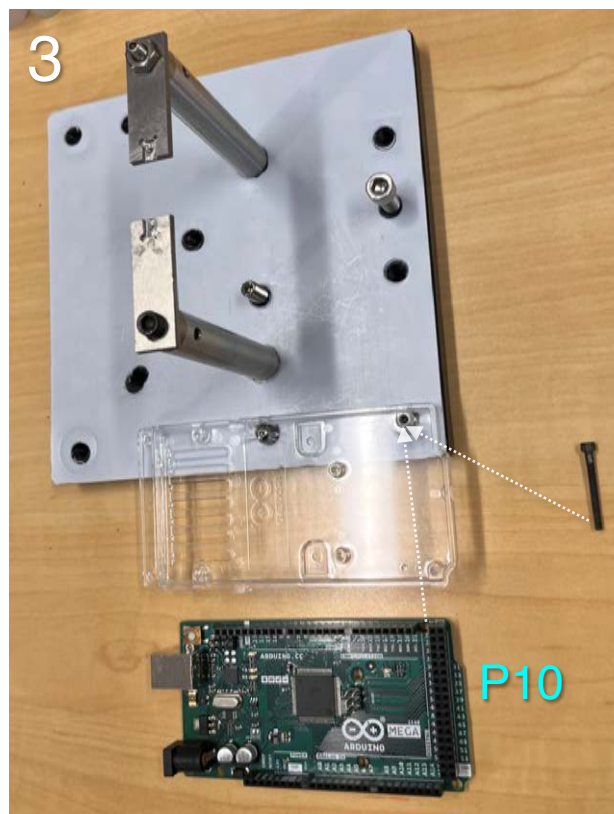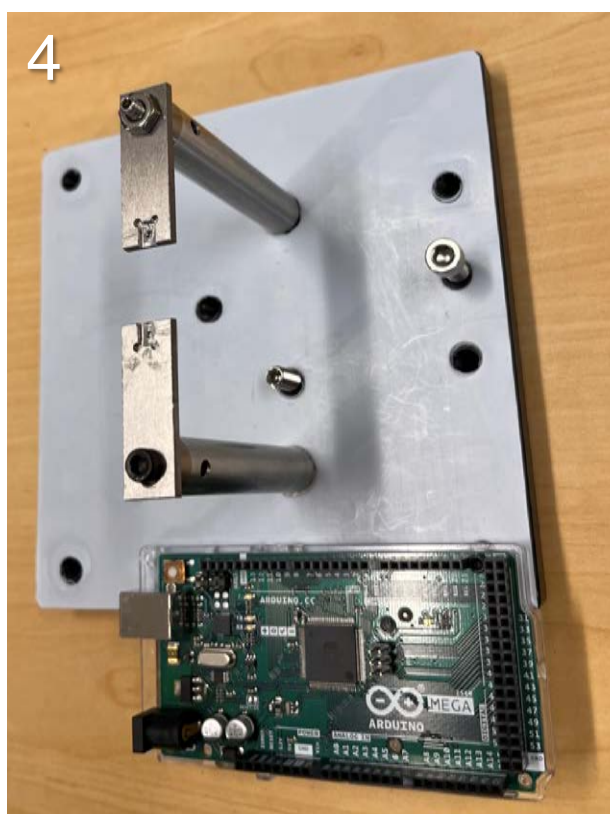

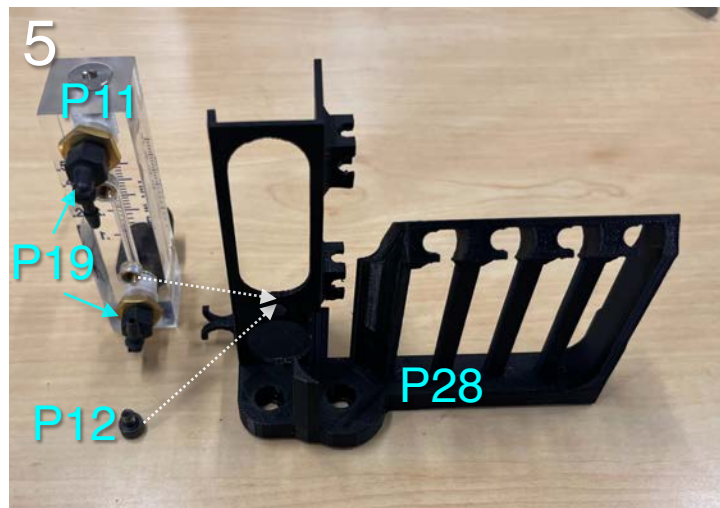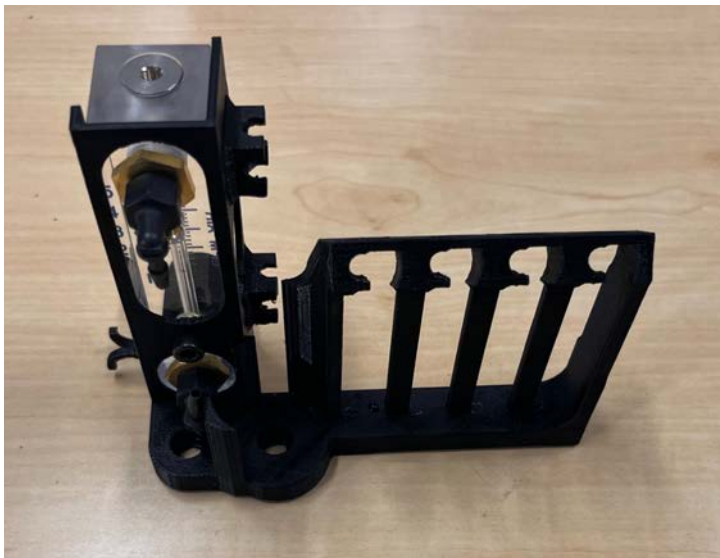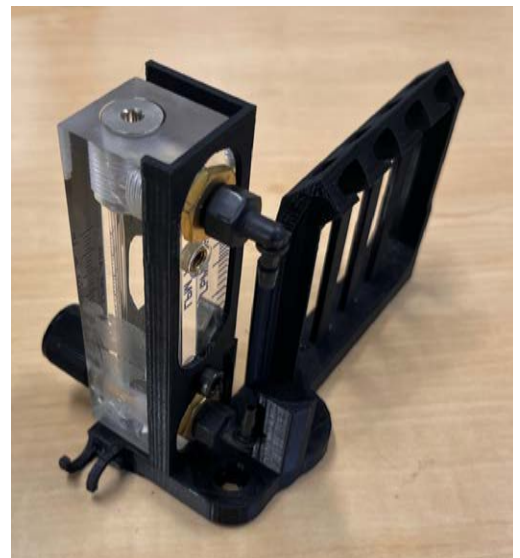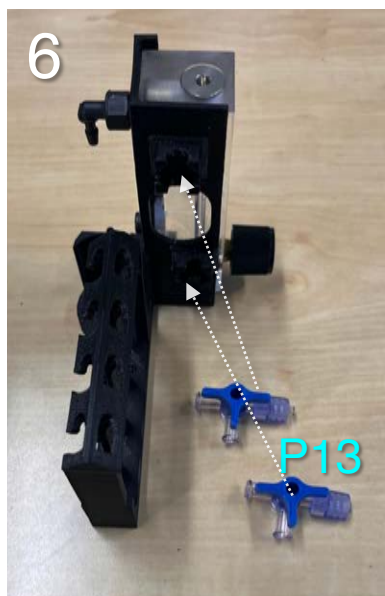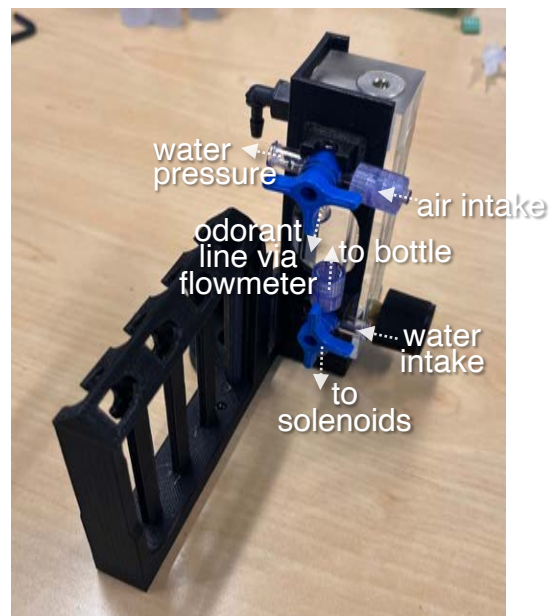

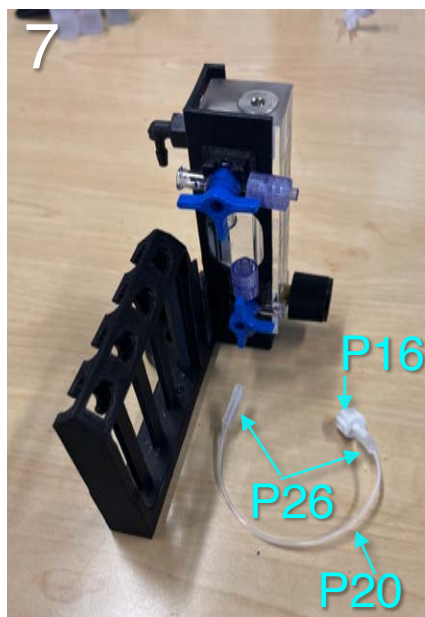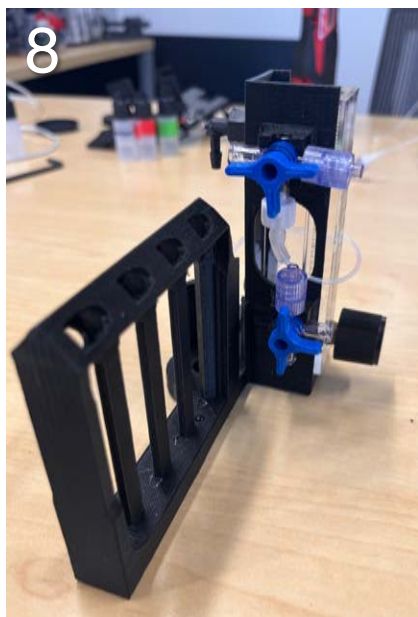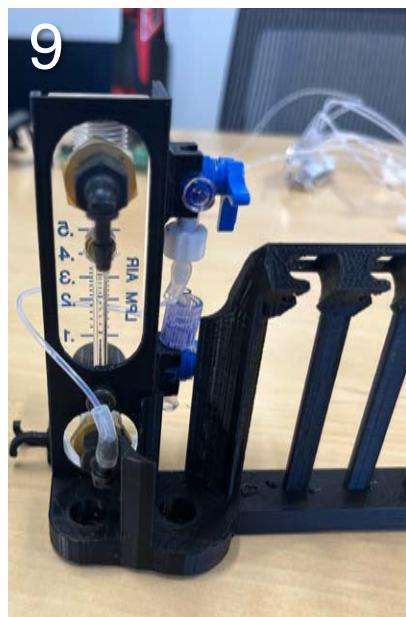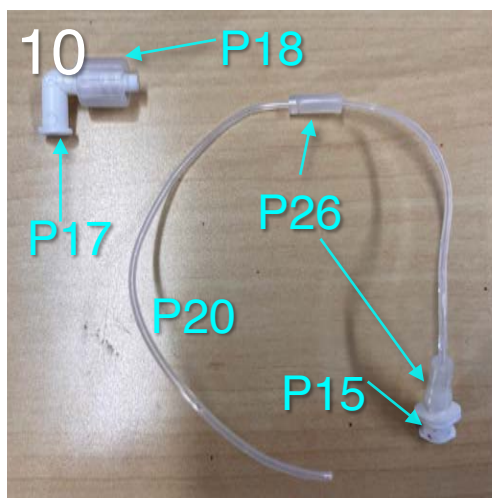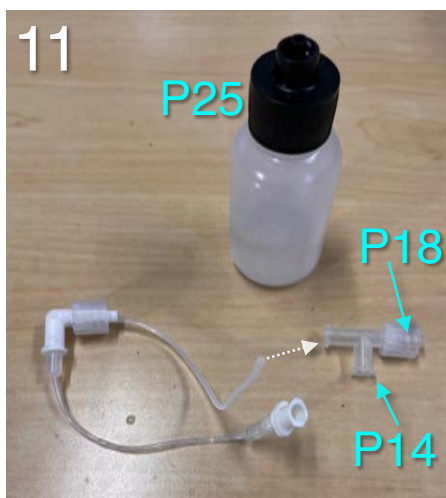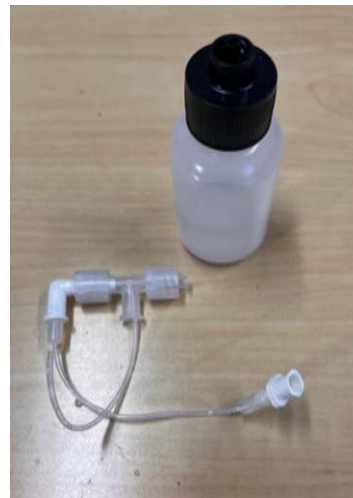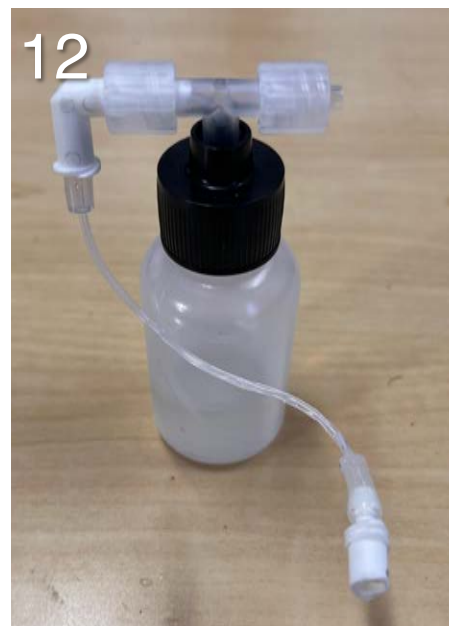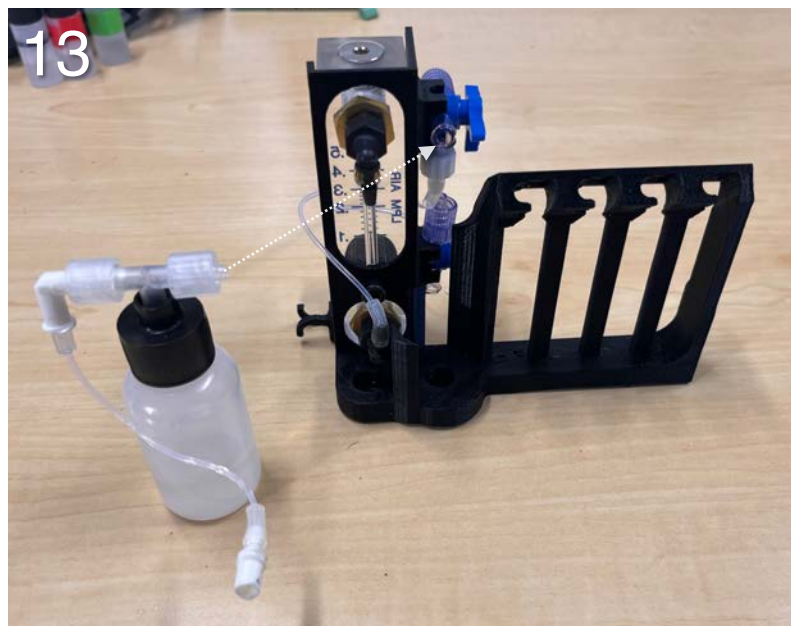

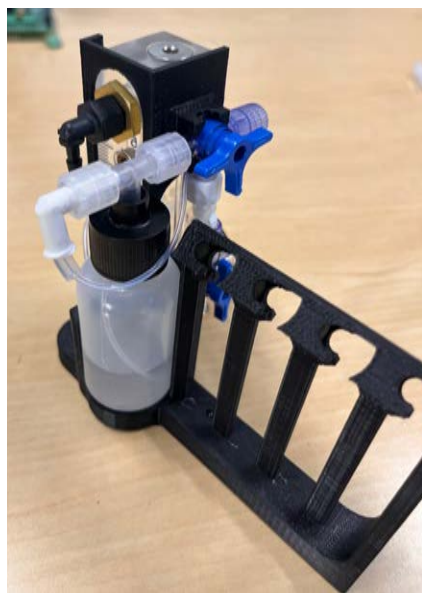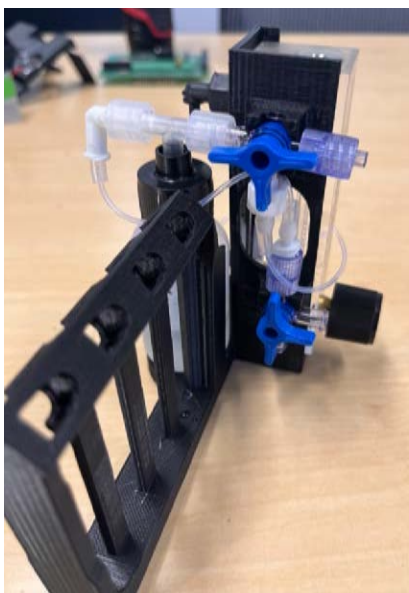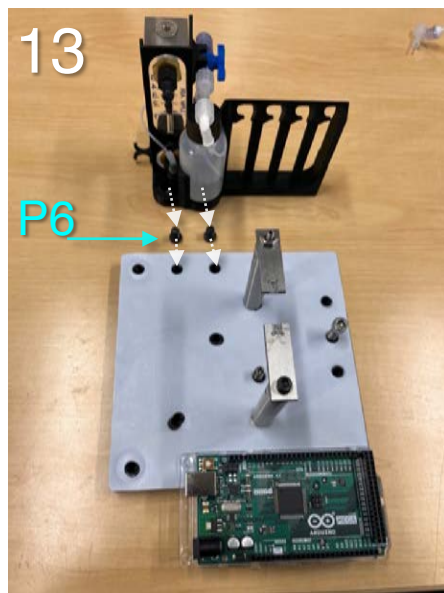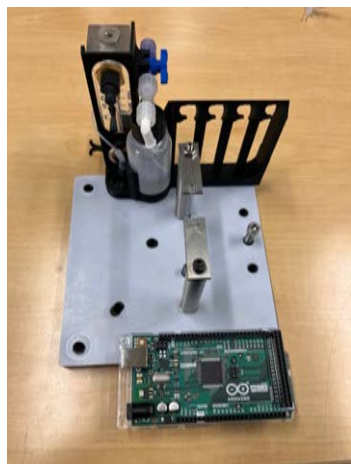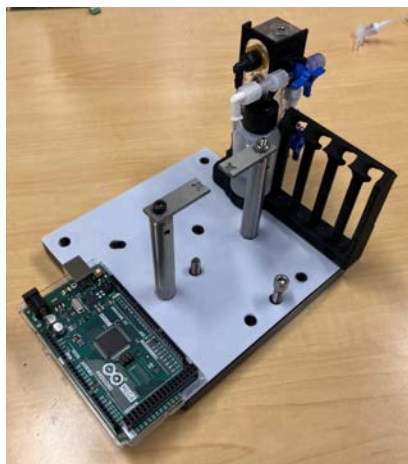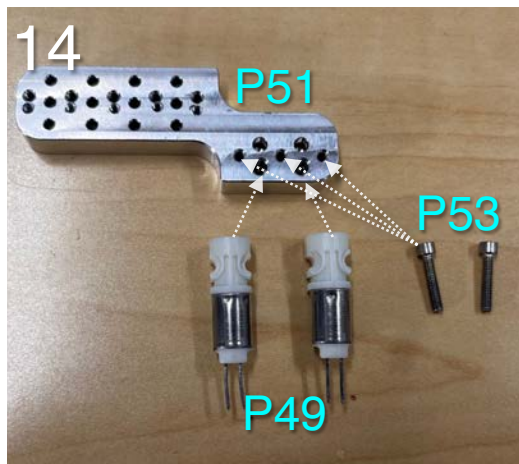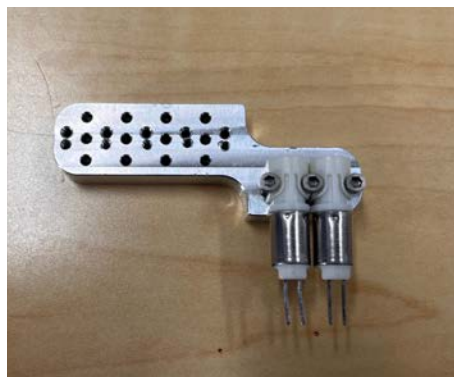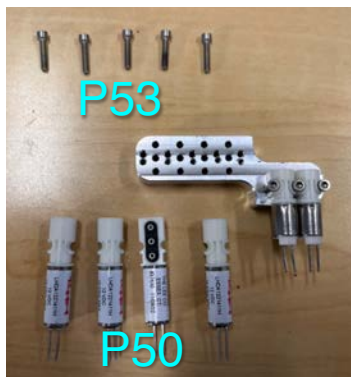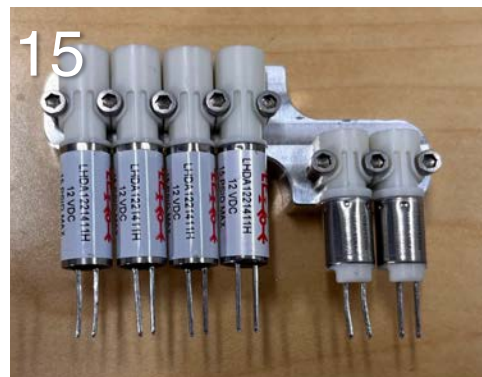

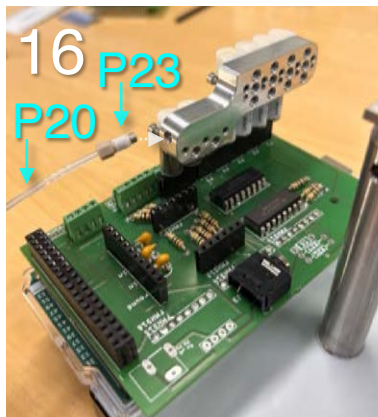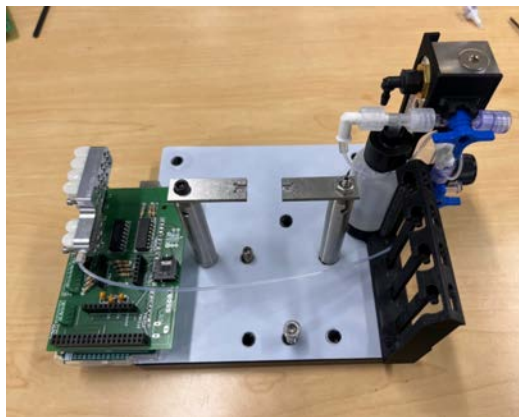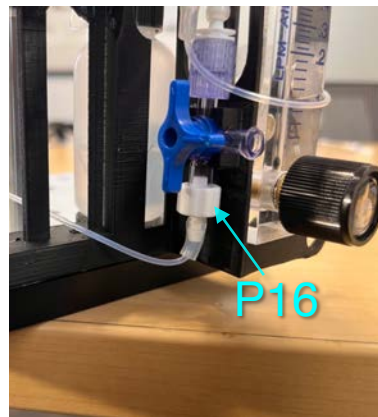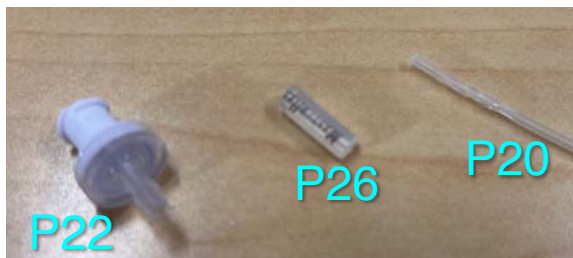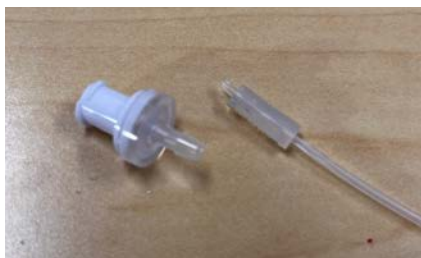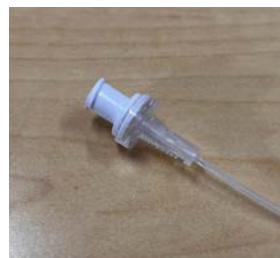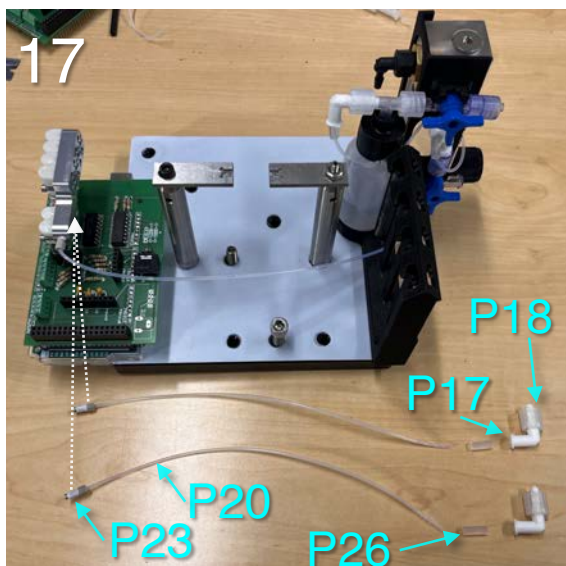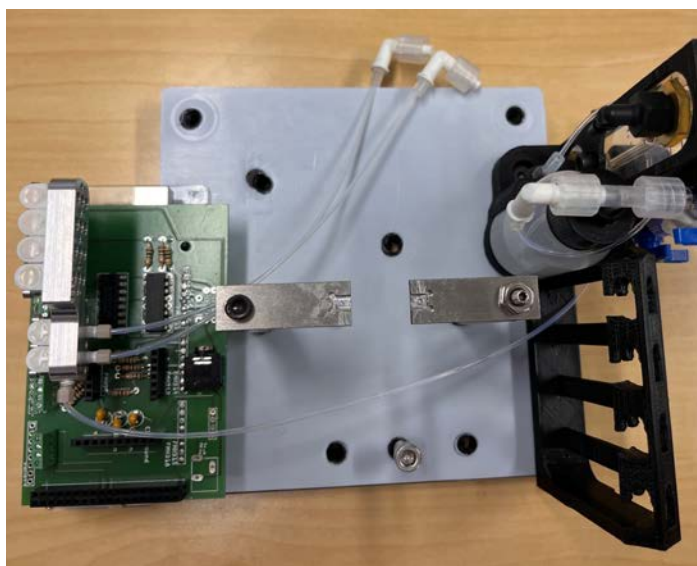

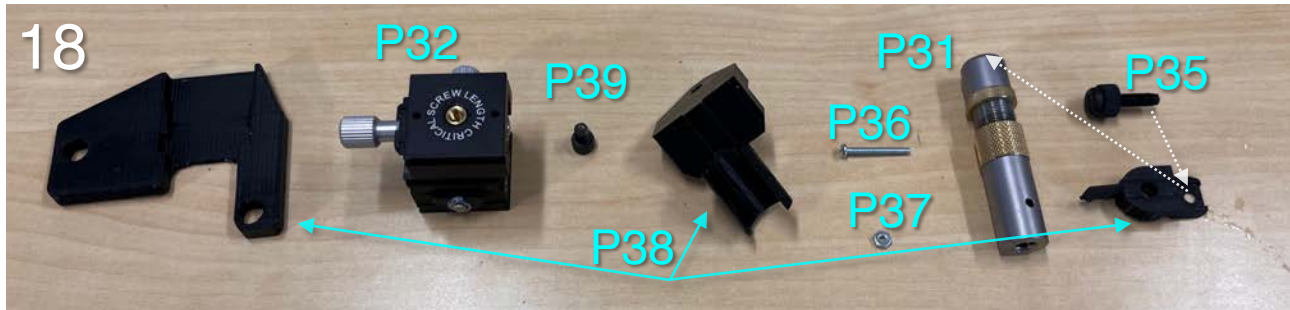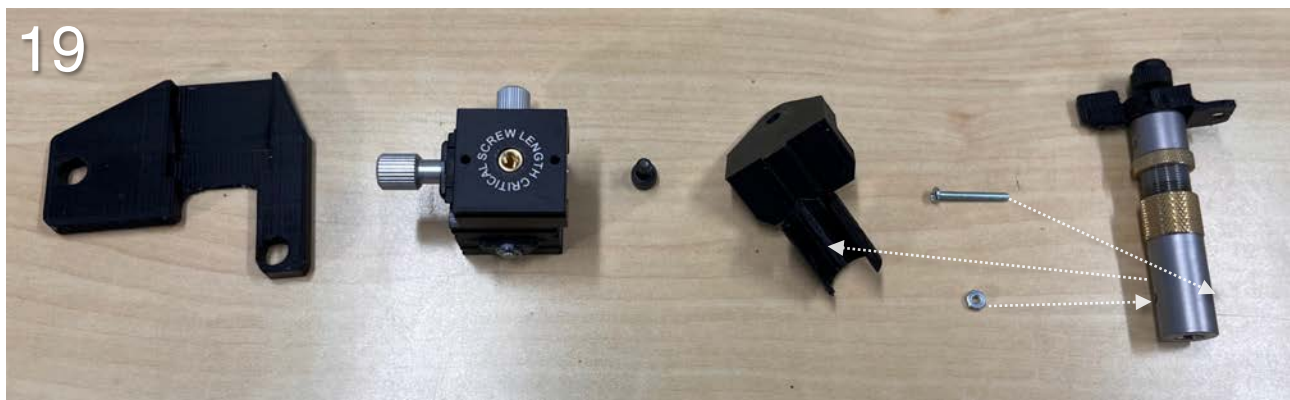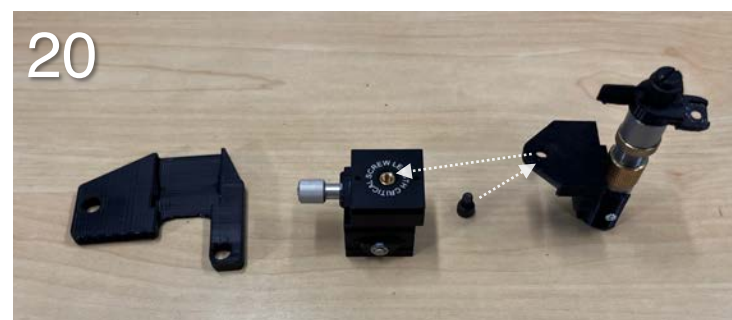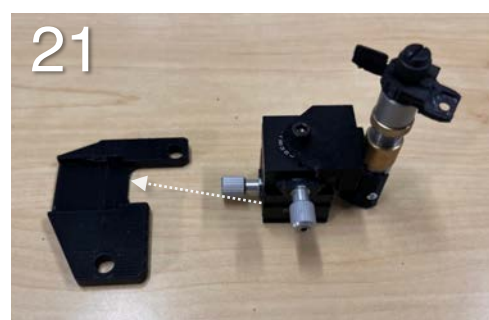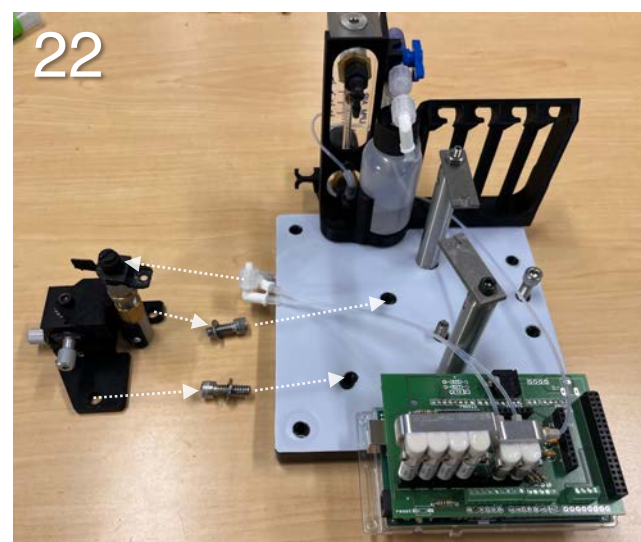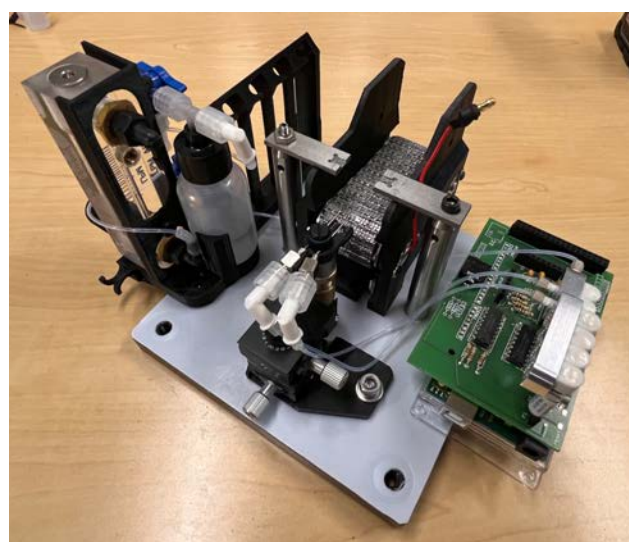

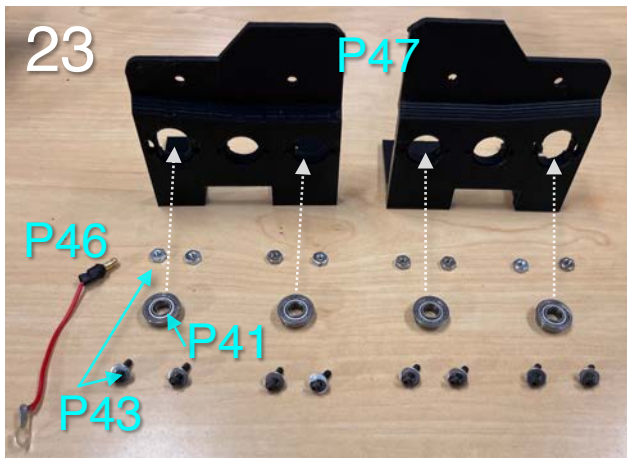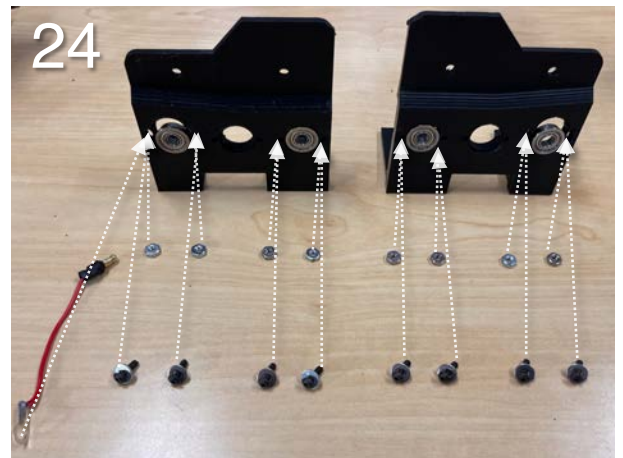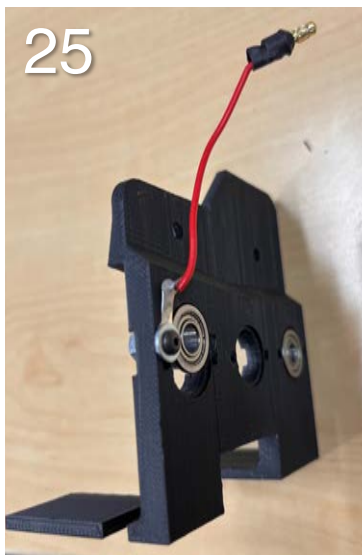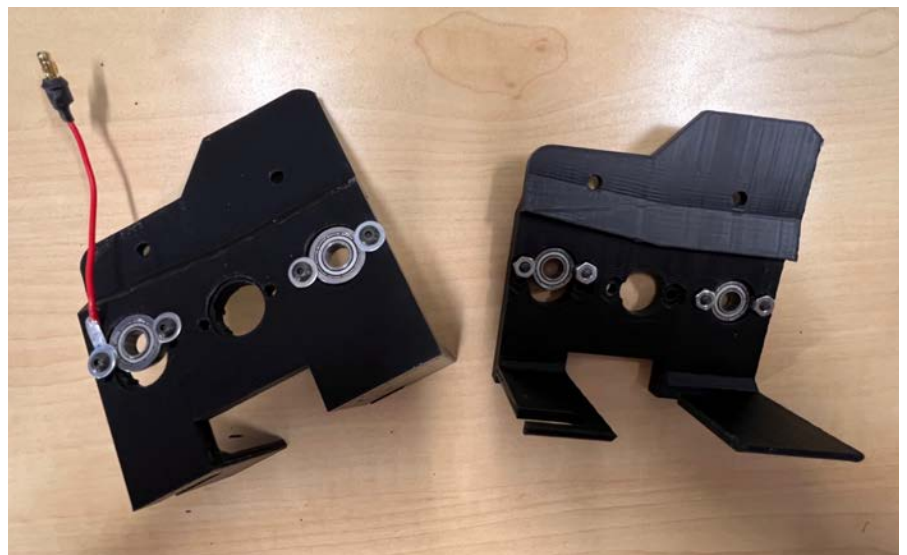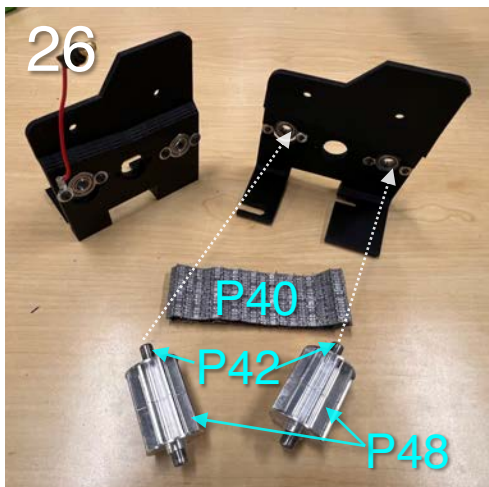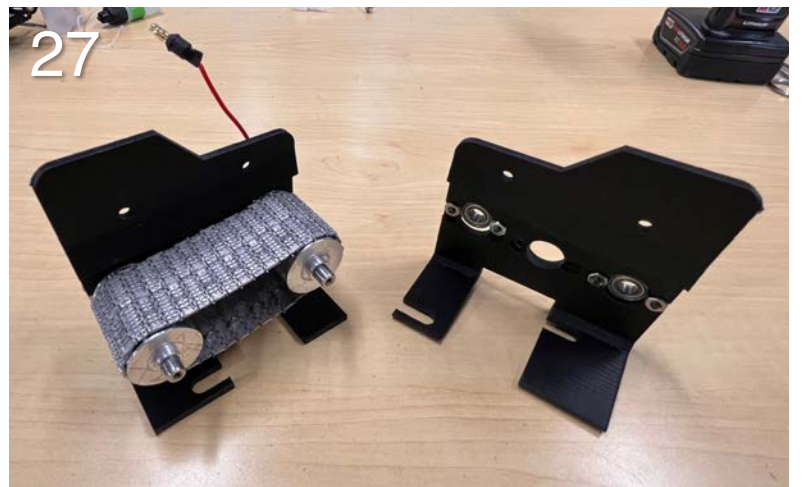

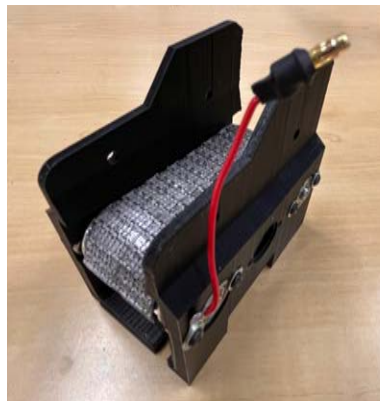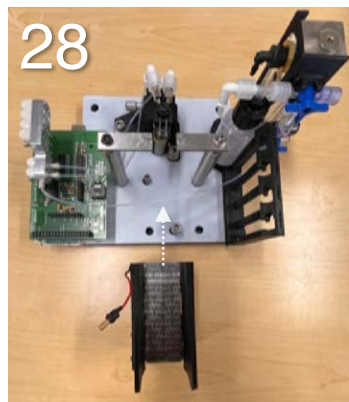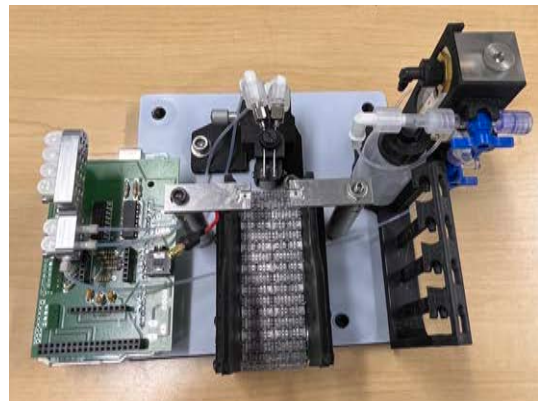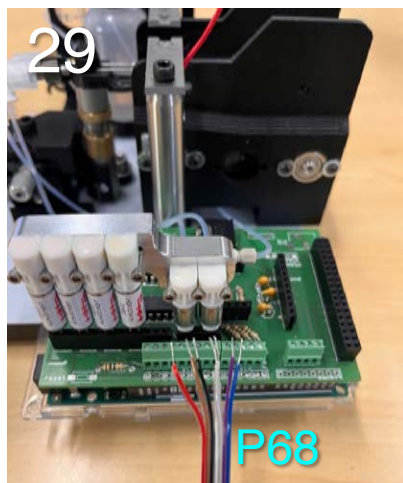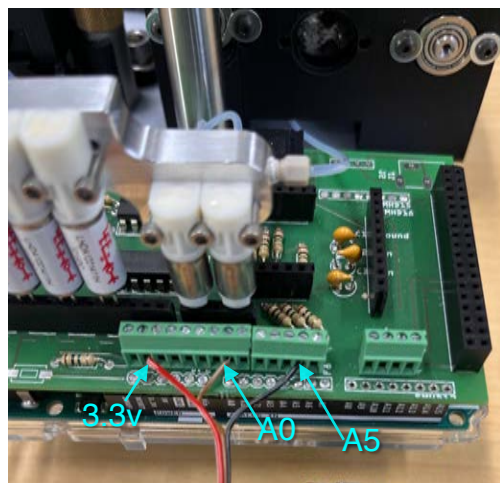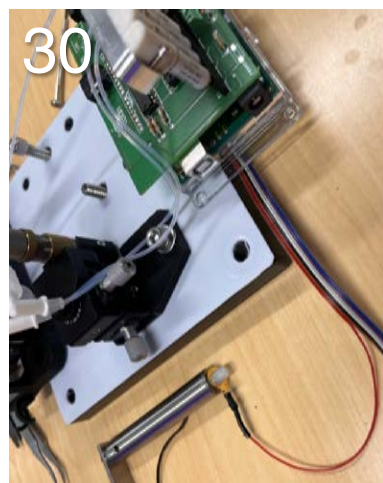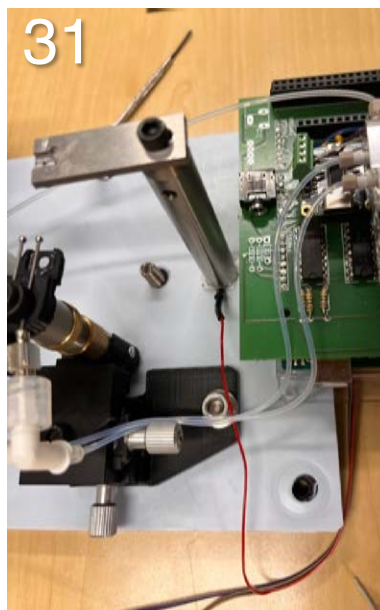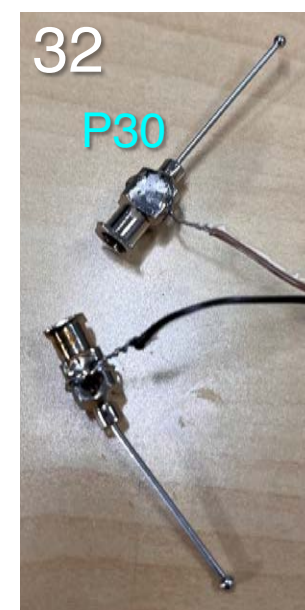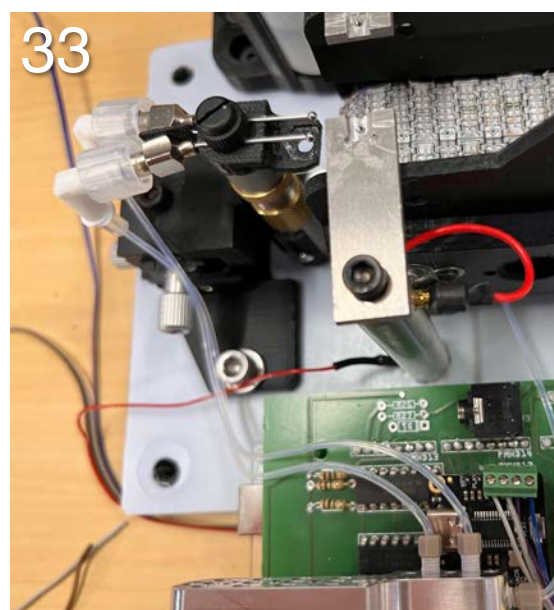

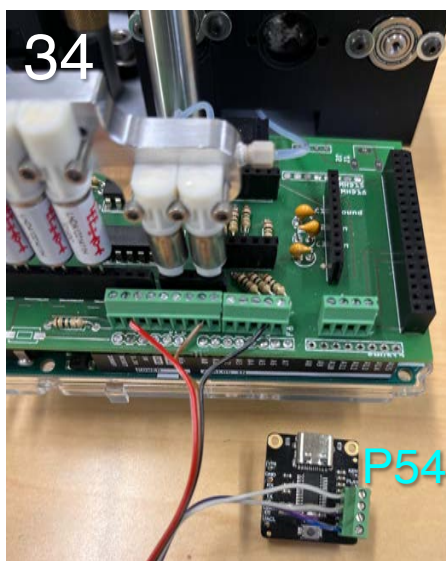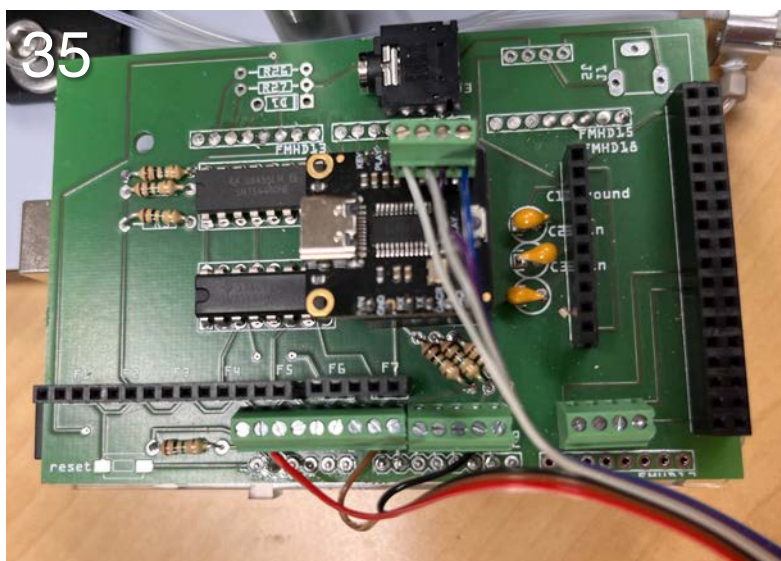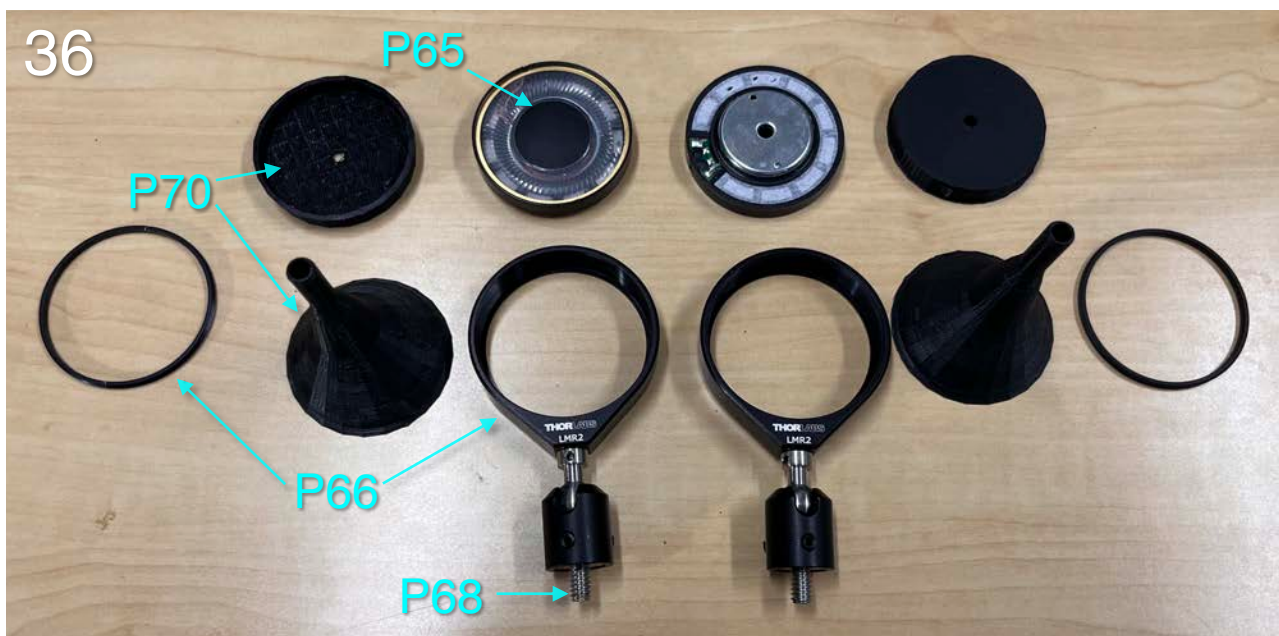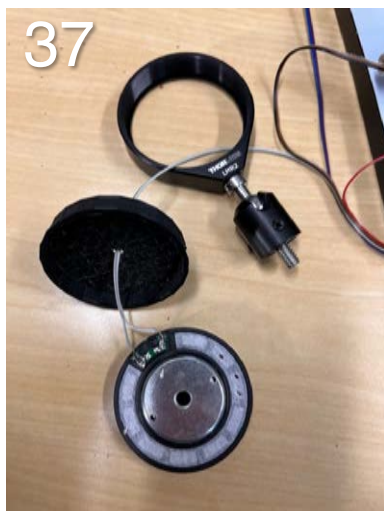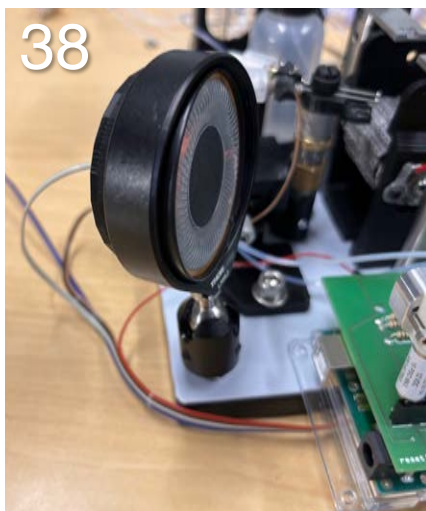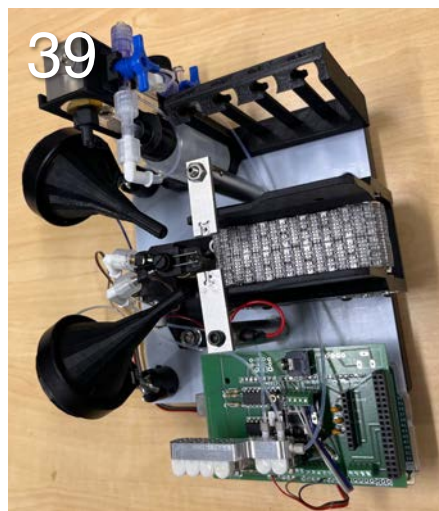

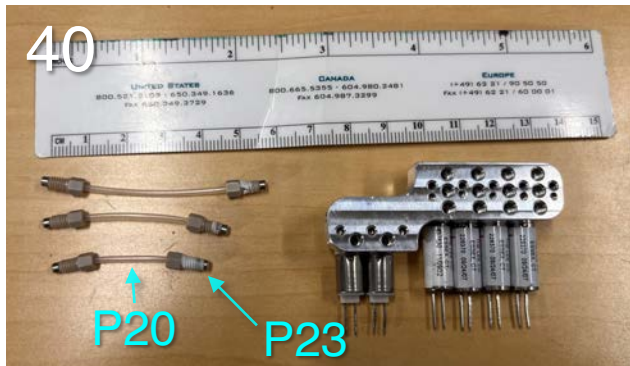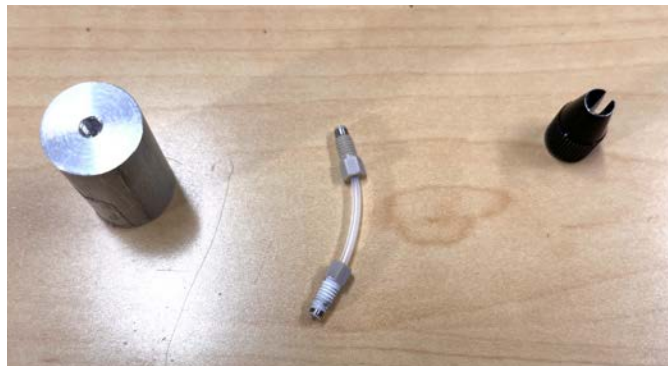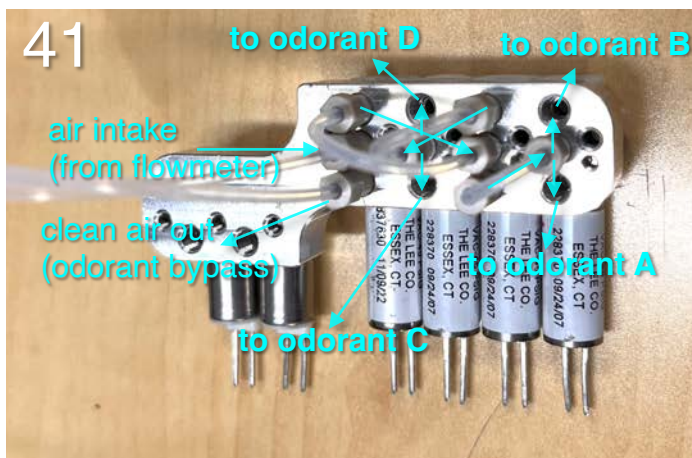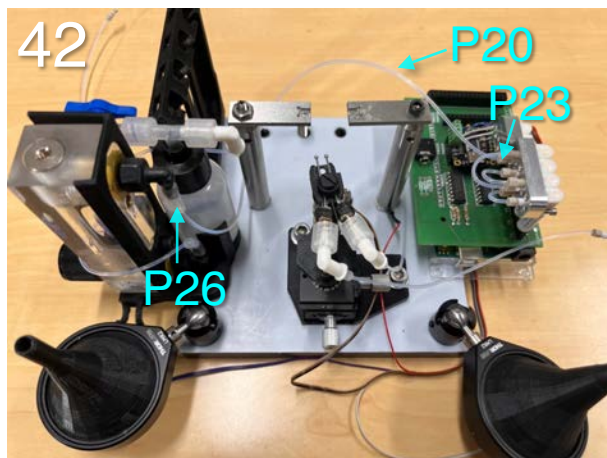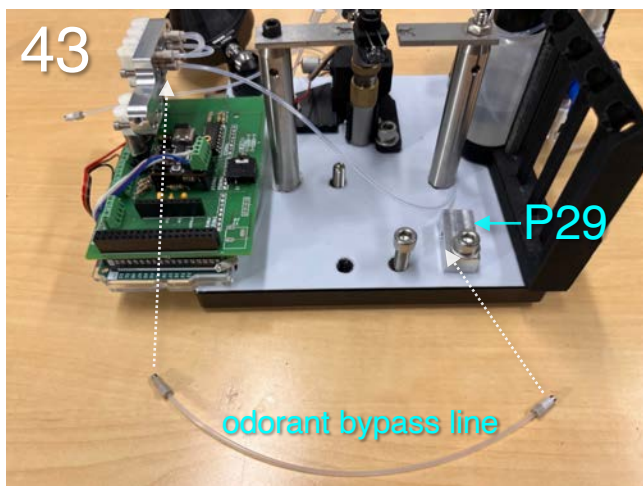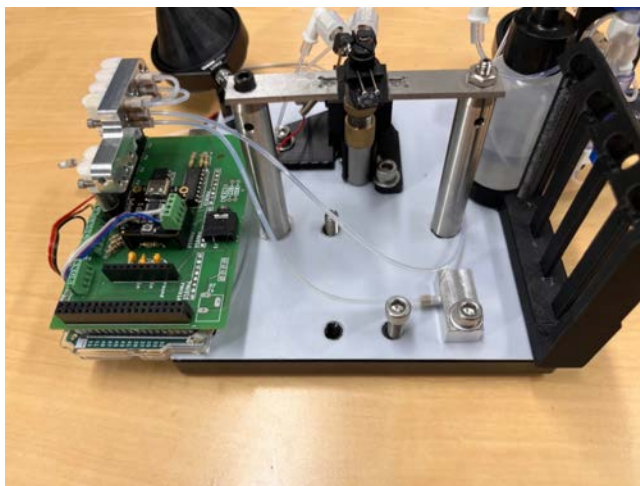

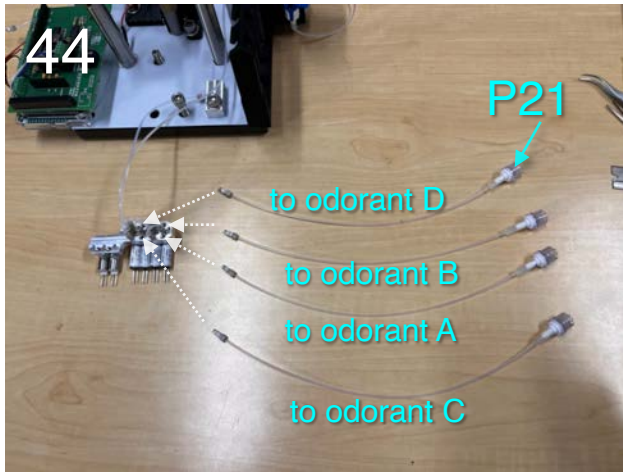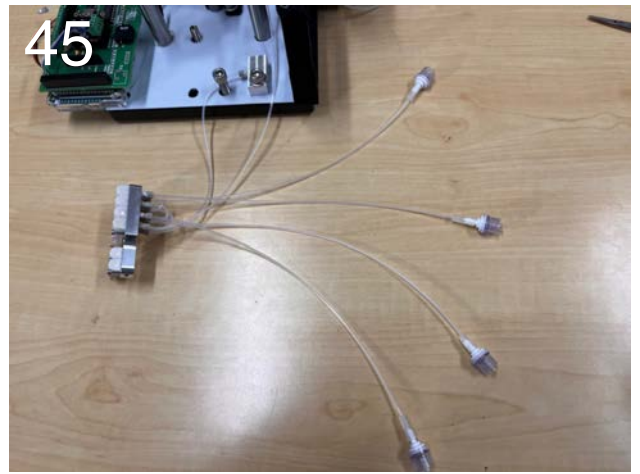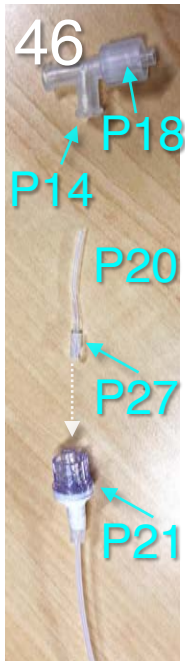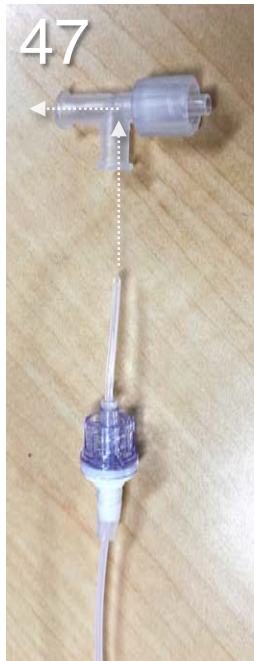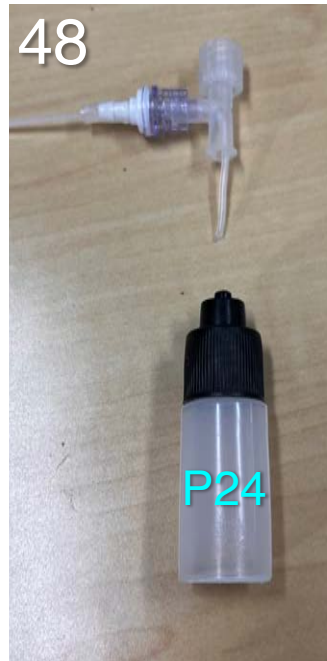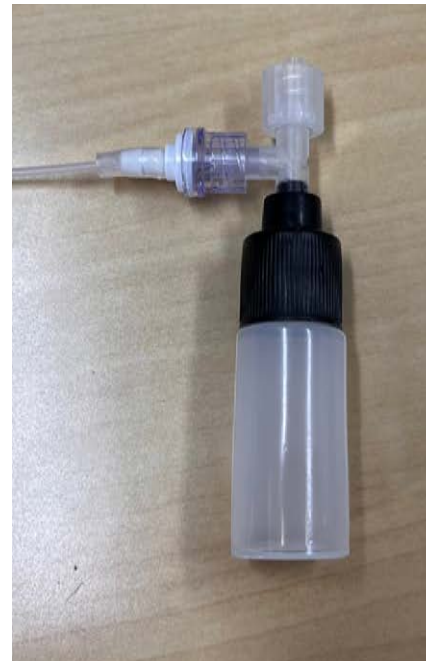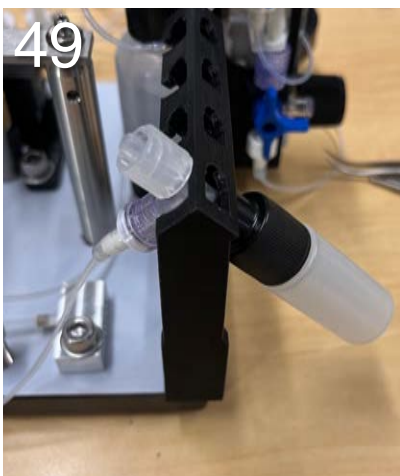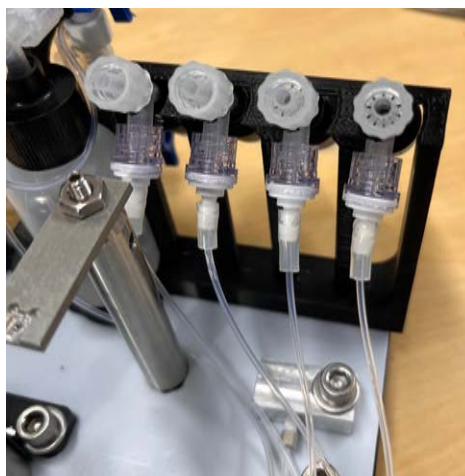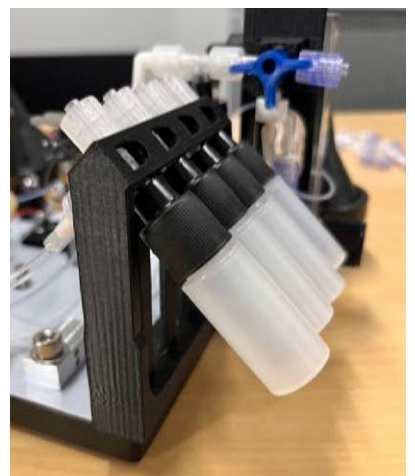

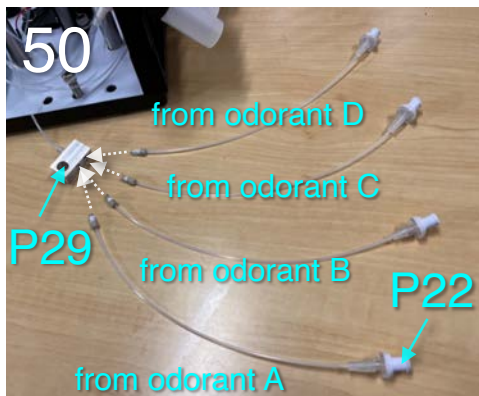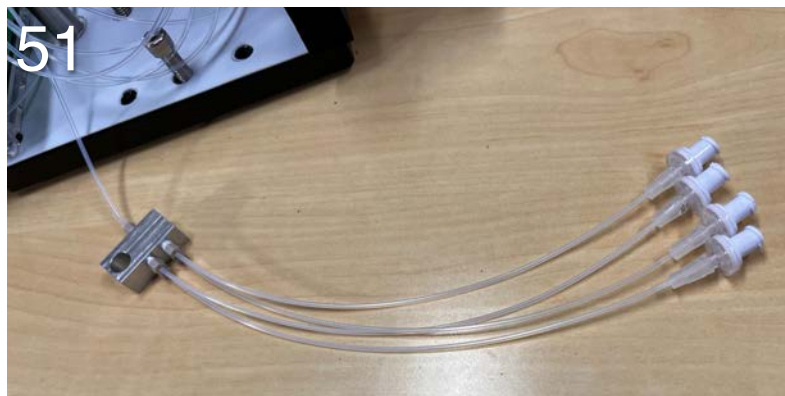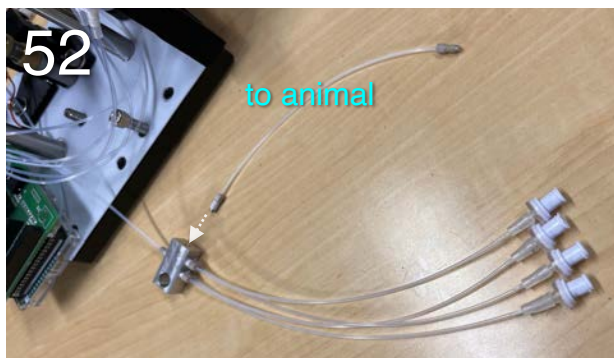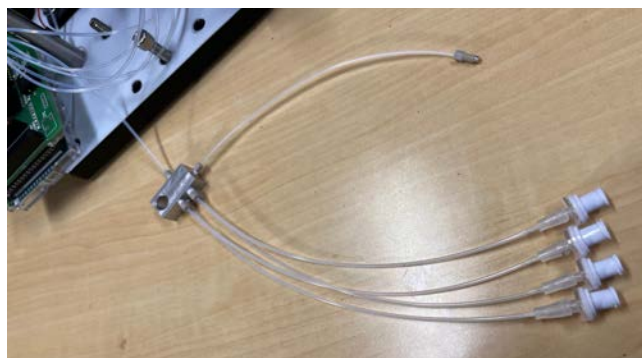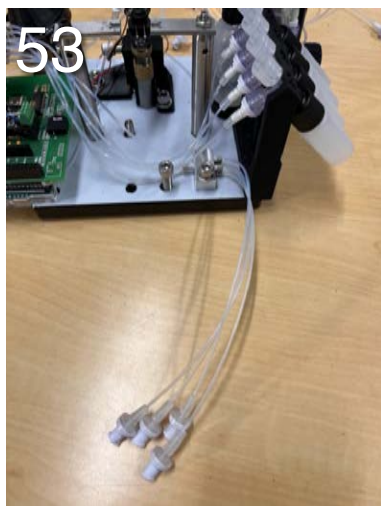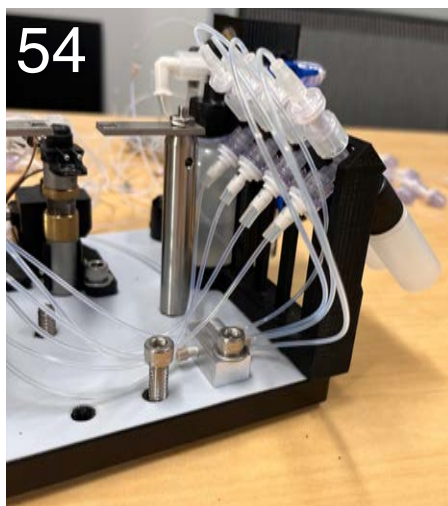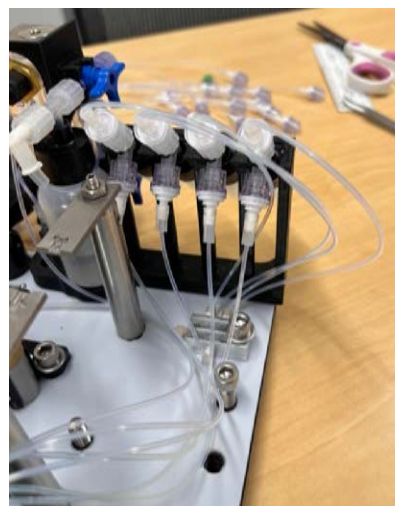

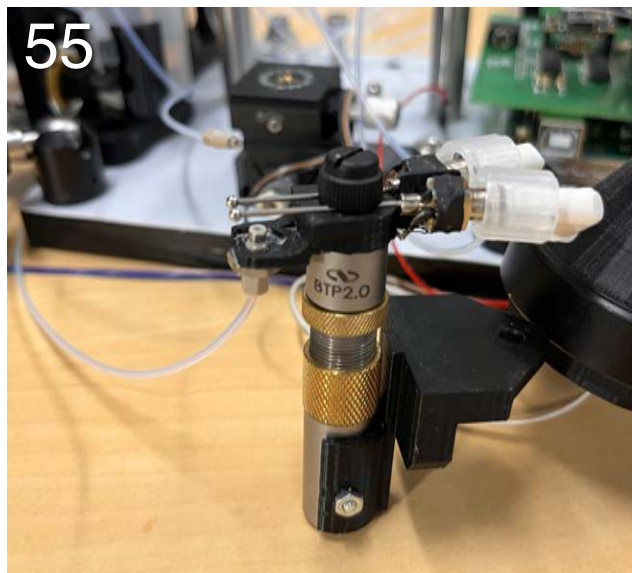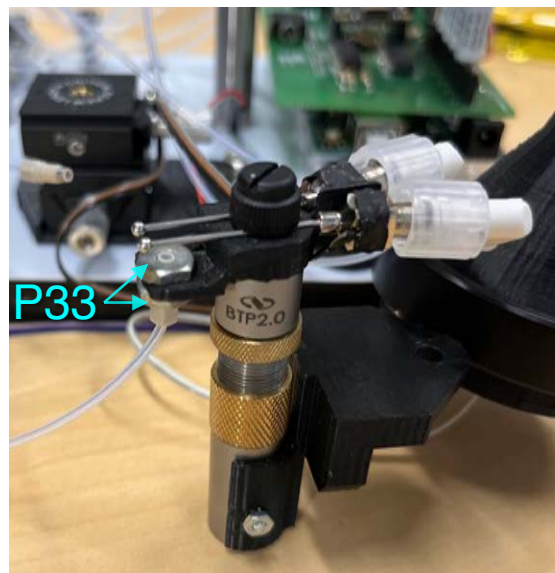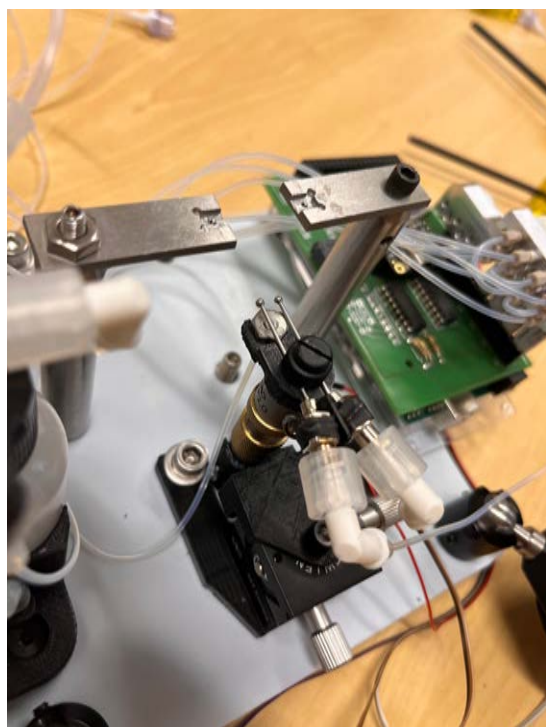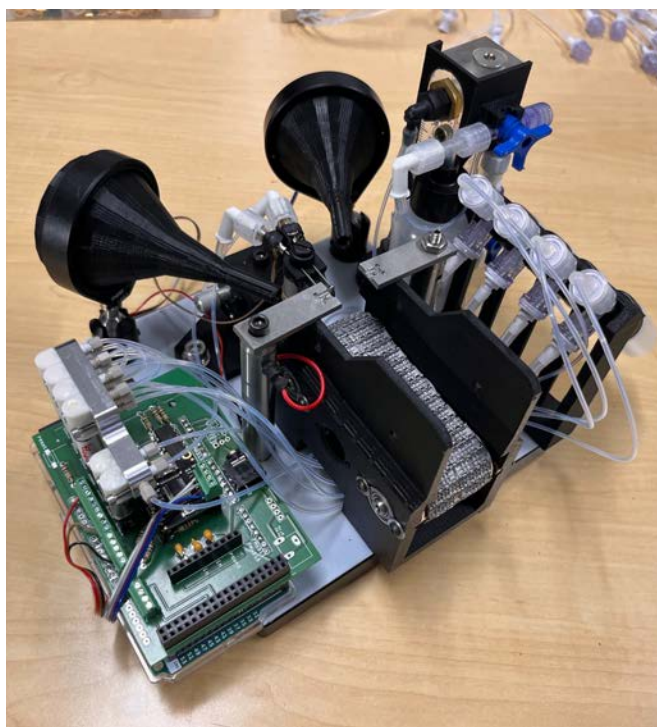

Supplement: FlexRig Repository — Download FlexRig Repository, ZIP file. [file eneuro-12-ENEURO.0364-24.2024-s003.zip › FlexRig-main/AssemblyInstructions.pdf]

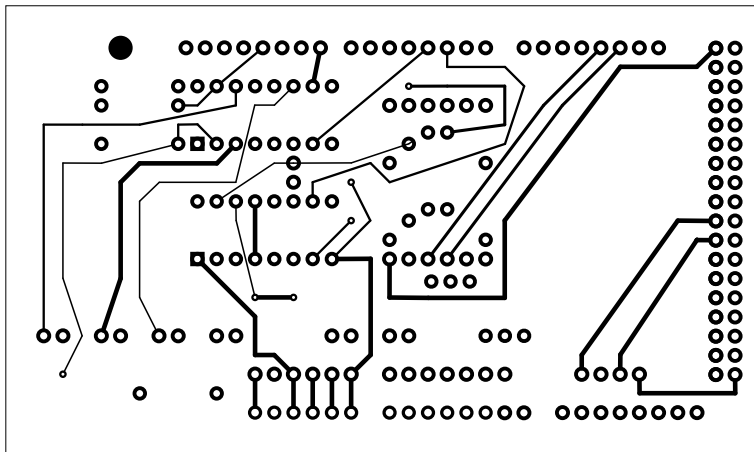

Supplement: FlexRig Repository — Download FlexRig Repository, ZIP file. [file eneuro-12-ENEURO.0364-24.2024-s003.zip › FlexRig-main/PCBFiles/FlexRigShield2_etch_copper_bottom.pdf]

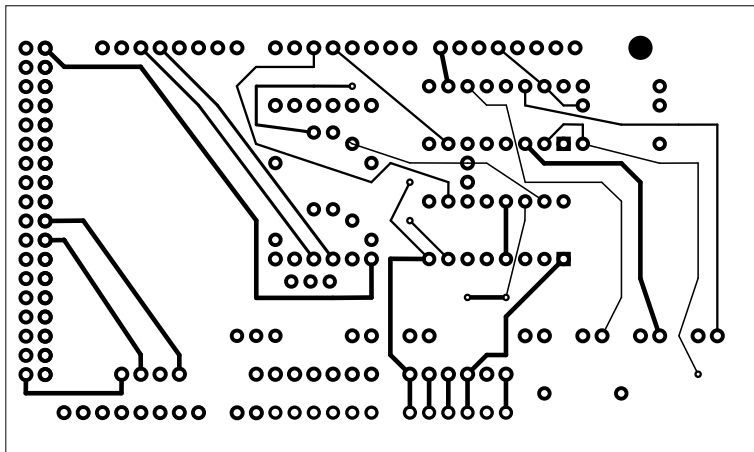

Supplement: FlexRig Repository — Download FlexRig Repository, ZIP file. [file eneuro-12-ENEURO.0364-24.2024-s003.zip › FlexRig-main/PCBFiles/FlexRigShield2_etch_copper_bottom_mirror.pdf]

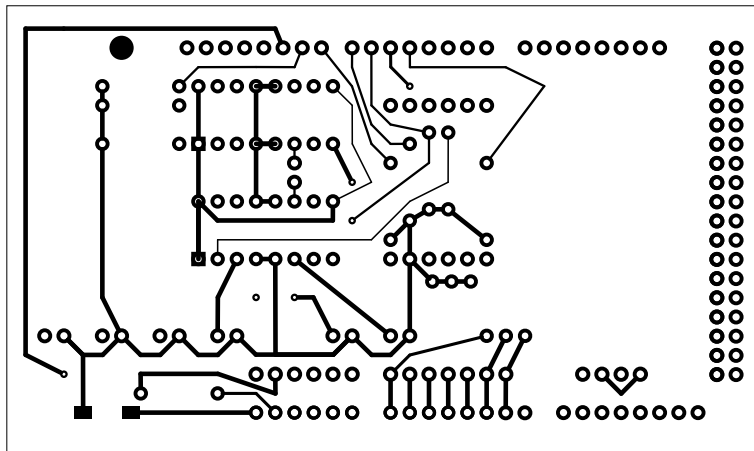

Supplement: FlexRig Repository — Download FlexRig Repository, ZIP file. [file eneuro-12-ENEURO.0364-24.2024-s003.zip › FlexRig-main/PCBFiles/FlexRigShield2_etch_copper_top.pdf]

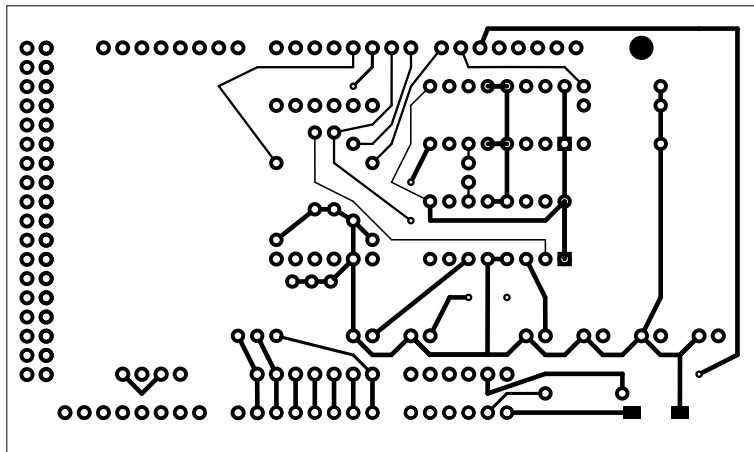

Supplement: FlexRig Repository — Download FlexRig Repository, ZIP file. [file eneuro-12-ENEURO.0364-24.2024-s003.zip › FlexRig-main/PCBFiles/FlexRigShield2_etch_copper_top_mirror.pdf]

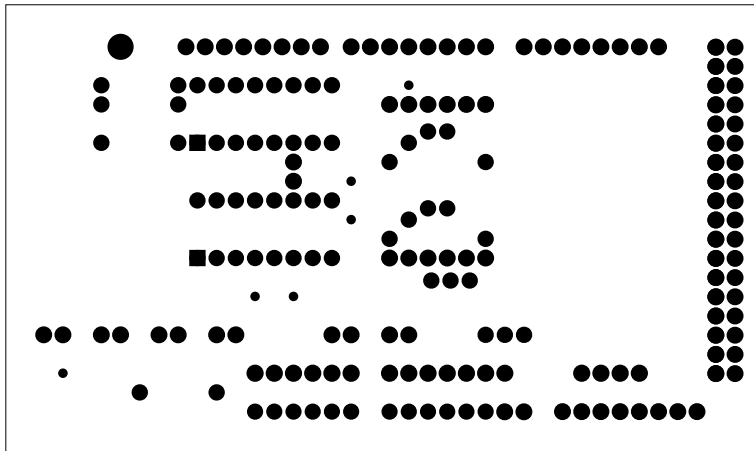

Supplement: FlexRig Repository — Download FlexRig Repository, ZIP file. [file eneuro-12-ENEURO.0364-24.2024-s003.zip › FlexRig-main/PCBFiles/FlexRigShield2_etch_mask_bottom.pdf]

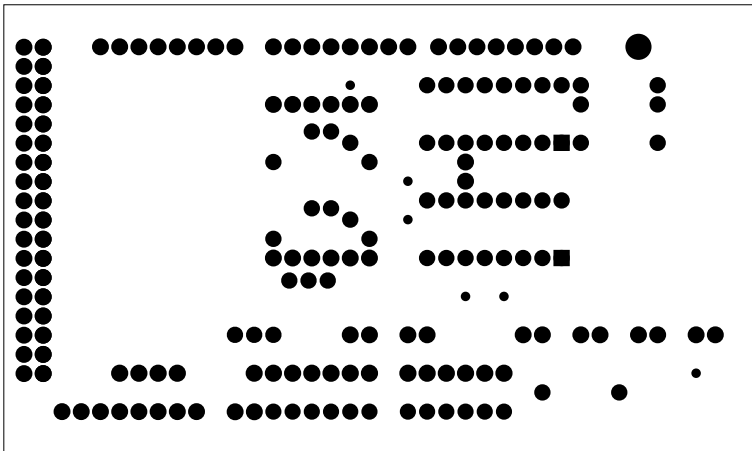

Supplement: FlexRig Repository — Download FlexRig Repository, ZIP file. [file eneuro-12-ENEURO.0364-24.2024-s003.zip › FlexRig-main/PCBFiles/FlexRigShield2_etch_mask_bottom_mirror.pdf]

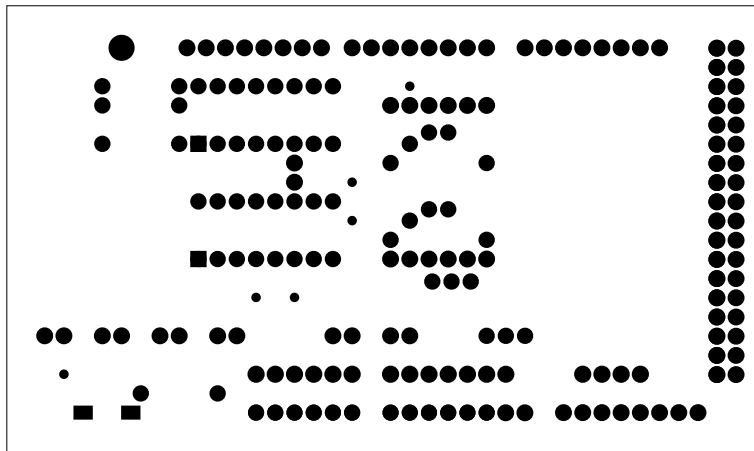

Supplement: FlexRig Repository — Download FlexRig Repository, ZIP file. [file eneuro-12-ENEURO.0364-24.2024-s003.zip › FlexRig-main/PCBFiles/FlexRigShield2_etch_mask_top.pdf]

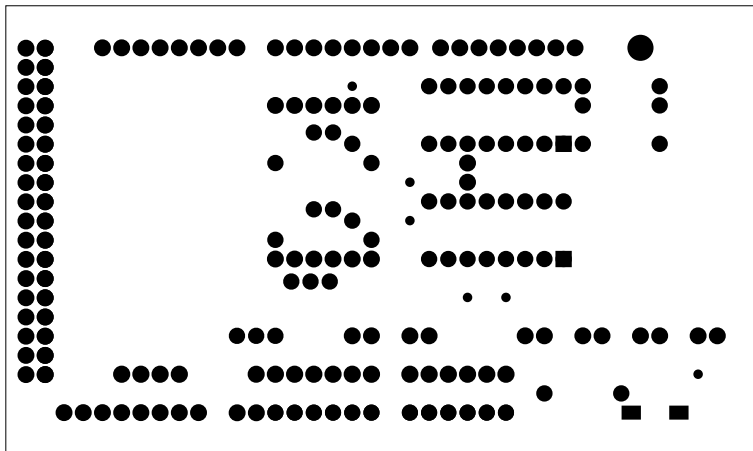

Supplement: FlexRig Repository — Download FlexRig Repository, ZIP file. [file eneuro-12-ENEURO.0364-24.2024-s003.zip › FlexRig-main/PCBFiles/FlexRigShield2_etch_mask_top_mirror.pdf]

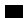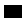

Supplement: FlexRig Repository — Download FlexRig Repository, ZIP file. [file eneuro-12-ENEURO.0364-24.2024-s003.zip › FlexRig-main/PCBFiles/FlexRigShield2_etch_paste_mask_top.pdf]

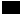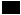

Supplement: FlexRig Repository — Download FlexRig Repository, ZIP file. [file eneuro-12-ENEURO.0364-24.2024-s003.zip › FlexRig-main/PCBFiles/FlexRigShield2_etch_paste_mask_top_mirror.pdf]

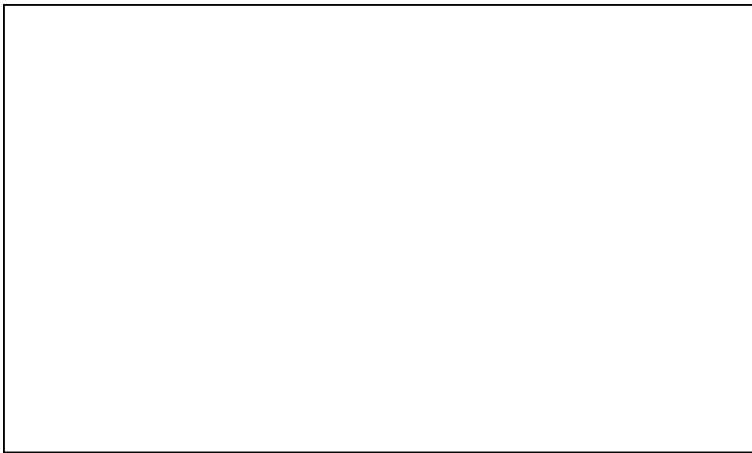

Supplement: FlexRig Repository — Download FlexRig Repository, ZIP file. [file eneuro-12-ENEURO.0364-24.2024-s003.zip › FlexRig-main/PCBFiles/FlexRigShield2_etch_silk_bottom.pdf]

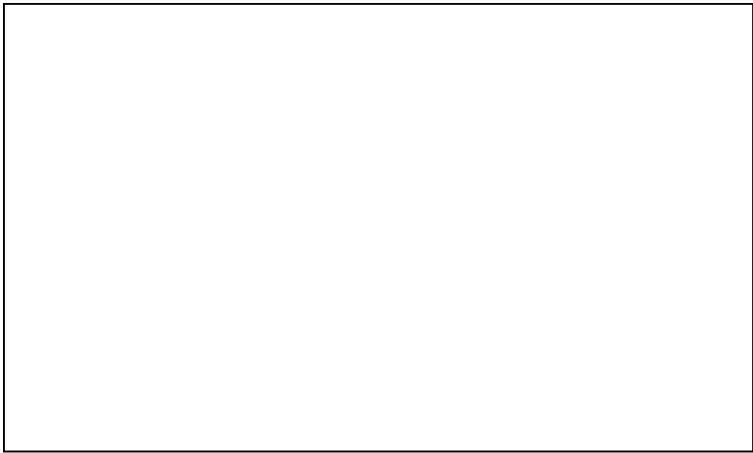

Supplement: FlexRig Repository — Download FlexRig Repository, ZIP file. [file eneuro-12-ENEURO.0364-24.2024-s003.zip › FlexRig-main/PCBFiles/FlexRigShield2_etch_silk_bottom_mirror.pdf]

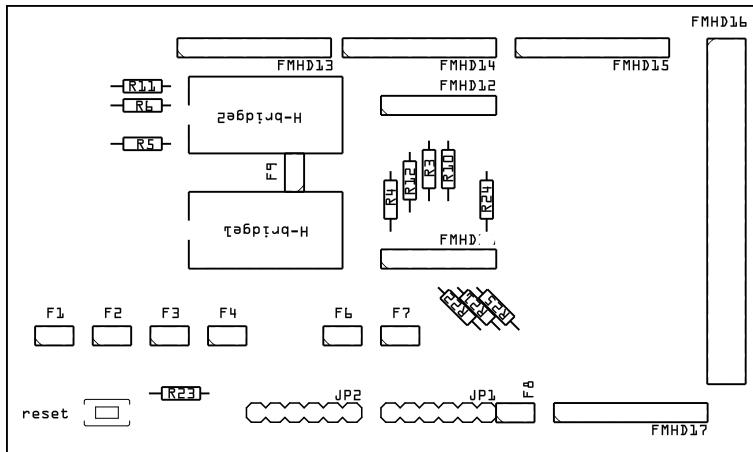

Supplement: FlexRig Repository — Download FlexRig Repository, ZIP file. [file eneuro-12-ENEURO.0364-24.2024-s003.zip › FlexRig-main/PCBFiles/FlexRigShield2_etch_silk_top.pdf]

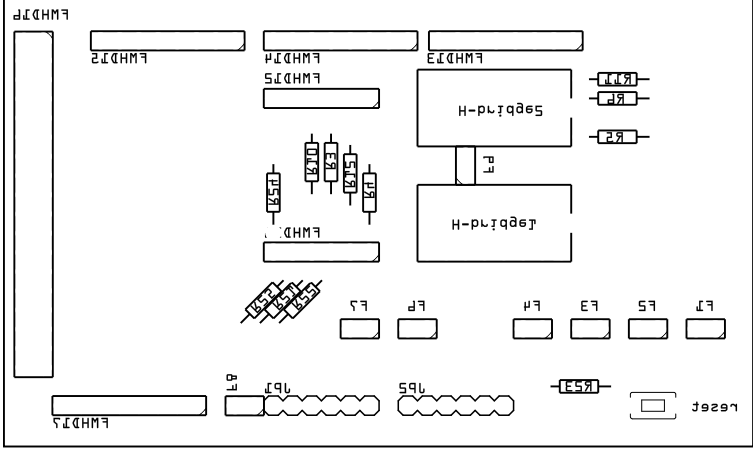

Supplement: FlexRig Repository — Download FlexRig Repository, ZIP file. [file eneuro-12-ENEURO.0364-24.2024-s003.zip › FlexRig-main/PCBFiles/FlexRigShield2_etch_silk_top_mirror.pdf]
